# Associations between the gut microbiome and metabolome in early life

**DOI:** 10.1186/s12866-021-02282-3

**Published:** 2021-08-28

**Authors:** Quang P. Nguyen, Margaret R. Karagas, Juliette C. Madan, Erika Dade, Thomas J. Palys, Hilary G. Morrison, Wimal W. Pathmasiri, Susan McRitche, Susan J. Sumner, H. Robert Frost, Anne G. Hoen

**Affiliations:** 1 Department of Epidemiology, Geisel School of Medicine at Dartmouth College, Hanover, NH USA; 2 Department of Biomedical Data Science, Geisel School of Medicine at Dartmouth College, Hanover, NH USA; 3 Children’s Environmental Health & Disease Prevention Research Center at Dartmouth, Lebanon, NH USA; 4 Department of Pediatrics, Children’s Hospital at Dartmouth, Hanover, NH USA; 5 Josephine Bay Paul Center, Marine Biological Laboratory, Woods Hole, MA USA; 6 Department of Nutrition, Nutrition Research Institute, University of North Carolina at Chapel Hill, Chapel Hill, NC USA

**Keywords:** Infant gut microbiome, Stool metabolome, Prediction models, Functional redundancy, Metabolism

## Abstract

**Background:**

The infant intestinal microbiome plays an important role in metabolism and immune development with impacts on lifelong health. The linkage between the taxonomic composition of the microbiome and its metabolic phenotype is undefined and complicated by redundancies in the taxon-function relationship within microbial communities. To inform a more mechanistic understanding of the relationship between the microbiome and health, we performed an integrative statistical and machine learning-based analysis of microbe taxonomic structure and metabolic function in order to characterize the taxa-function relationship in early life.

**Results:**

Stool samples collected from infants enrolled in the New Hampshire Birth Cohort Study (NHBCS) at approximately 6-weeks (*n* = 158) and 12-months (*n* = 282) of age were profiled using targeted and untargeted nuclear magnetic resonance (NMR) spectroscopy as well as DNA sequencing of the V4-V5 hypervariable region from the bacterial 16S rRNA gene. There was significant inter-omic concordance based on Procrustes analysis (6 weeks: *p* = 0.056; 12 months: *p* = 0.001), however this association was no longer significant when accounting for phylogenetic relationships using generalized UniFrac distance metric (6 weeks: *p* = 0.376; 12 months: *p* = 0.069). Sparse canonical correlation analysis showed significant correlation, as well as identifying sets of microbe/metabolites driving microbiome-metabolome relatedness. Performance of machine learning models varied across different metabolites, with support vector machines (radial basis function kernel) being the consistently top ranked model. However, predictive R^2^ values demonstrated poor predictive performance across all models assessed (avg: − 5.06% -- 6 weeks; − 3.7% -- 12 months). Conversely, the Spearman correlation metric was higher (avg: 0.344–6 weeks; 0.265–12 months). This demonstrated that taxonomic relative abundance was not predictive of metabolite concentrations.

**Conclusions:**

Our results suggest a degree of overall association between taxonomic profiles and metabolite concentrations. However, lack of predictive capacity for stool metabolic signatures reflects, in part, the possible role of functional redundancy in defining the taxa-function relationship in early life as well as the bidirectional nature of the microbiome-metabolome association. Our results provide evidence in favor of a multi-omic approach for microbiome studies, especially those focused on health outcomes.

**Supplementary Information:**

The online version contains supplementary material available at 10.1186/s12866-021-02282-3.

## Background

The human gut microbiome is a complex and diverse system of microorganisms that co-inhabit the intestinal lumen and play a crucial role in modulating human health and disease [1, 2]. The development of the microbiota in early life is a sensitive process akin to primary ecological succession [3], and therefore both reliant on, and vulnerable to, external perturbations. Studies have linked microbiome alterations to long-term health consequences, including risk of obesity [4], type I diabetes [5], and inflammatory bowel disease [6]. As such, there is a need to understand how the microbiome participates in the multifactorial pathways leading to long-term disease outcomes. One key to this open question lies in the currently undefined relationship between the taxonomic structure of the microbiome and its metabolic phenotype. Previous studies addressing this question have mainly focused on DNA-based profiling of microbial functional potential, which, due to complicated regulatory mechanisms at the cellular level beyond the genome, is not equivalent to the microbiota’s realized functional landscape [7].

There exists a bidirectional association between the metabolome and the microbiome in the gut [8, 9]. These low molecular weight molecules can either be nutrients that shape the composition of the microbiome [10] or important byproducts of host-microbe nutrient co-metabolism that help regulate host metabolic homeostasis [11–13]. For example, members of the *Clostridium* clusters can produce and increase serum levels of branched chain amino acids, which are markers for insulin resistance and diabetes [14, 15]. However, studies suggest that the fecal metabolome specifically can be used as a readout of gut microbe metabolic functions. Zierer et al. [16] showed, in a large cohort of adult females (*n* = 786) from the TwinsUK study, that around 60% of the fecal metabolome is associated with microbial composition, where on average, 67% of variance in the metabolome can be explained by the microbiome.

Recent studies have integrated the metabolome in microbiome analyses of health outcomes, most notably Lloyd et al. [17] from the integrative Human Microbiome Project. However, these studies have mostly focused on adult populations with specific metabolic disease etiologies such as inflammatory bowel disease. Only a limited number of studies [18–23] have simultaneously profiled the gut microbiome and metabolome from infant stool samples. These studies have preliminarily established that metabolomic profiles shift as taxonomic abundances change between subject case/control status [18, 20, 21, 24]. Specifically, Ayeni et al. (*n* = 48) [19] and Kisuse et al. (*n* = 35) [23] demonstrated that inter-sample distances calculated using metabolite abundances are correlated with those calculated from taxonomic profiles using Mantel tests across African and Asian cohorts. However, studies to date have either focused on preterm infants [18, 20, 21] or had small sample sizes (less than 50) [19, 22, 23]. We identified a major gap in defining microbiome-metabolome relatedness among infants from a population-based cohort capturing both early in infancy and near the first birthday, with regards to determining the strength of association and to identify key contributors to the overall concordance.

Here, we present our study investigating associations between microbe abundances assayed with 16S rRNA sequencing and metabolomic profiles measured with ^1^H NMR spectroscopy in a cohort of infants representing a rural general population from the New Hampshire Birth Cohort Study (NHBCS). This is a unique epidemiological cohort with multi-omic profiling of infant stool samples at multiple time points accompanied with rich sociodemographic, dietary and health outcomes data [25]. Our study utilizes predictive modeling, multivariate correlation methods and distance-based approaches to characterize the dynamic relationship between the gut microbiome and the gut metabolome in early life.

## Results

**Fig. 1:**
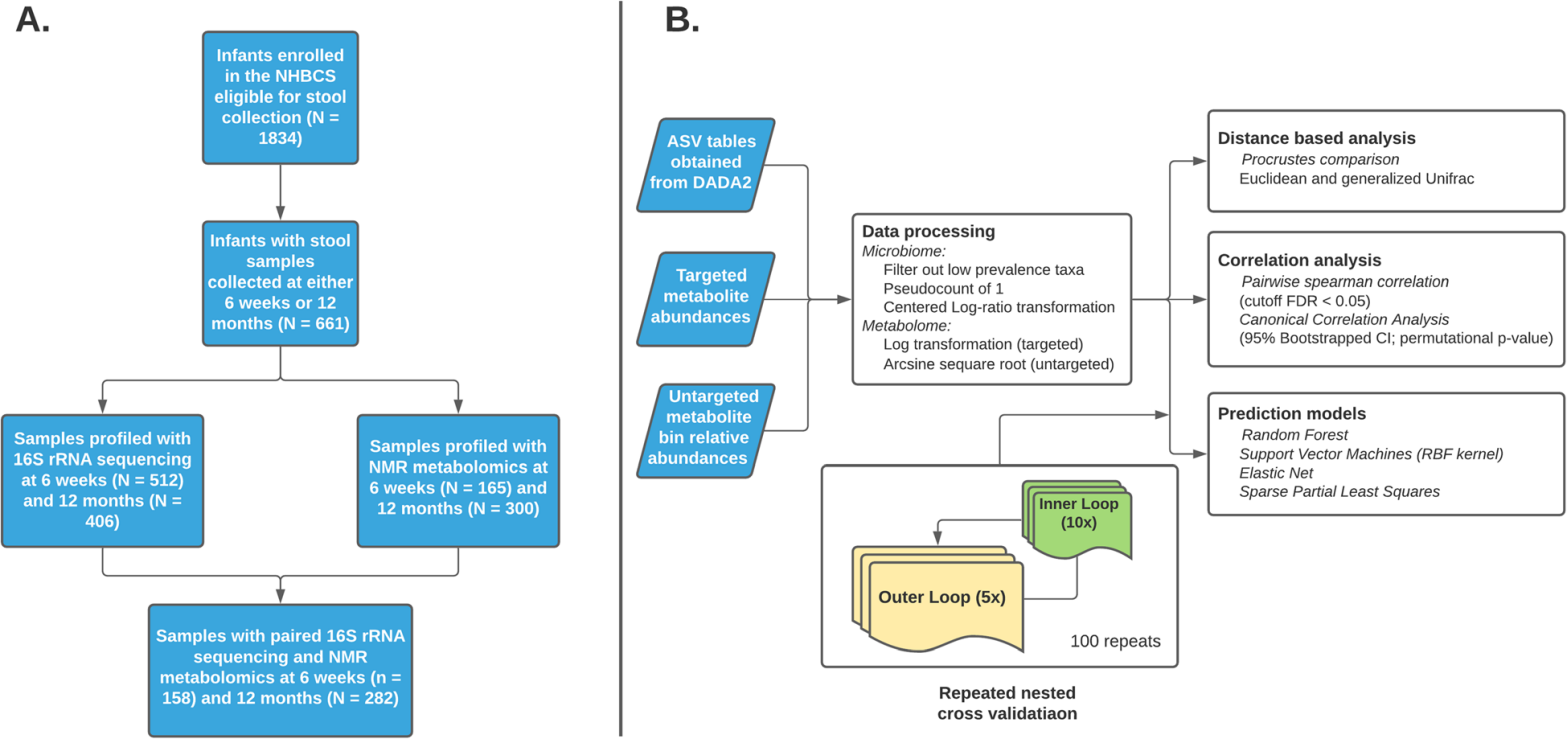
Overview of the analysis. Panel A describes the subject selection workflow and panel B describes the analytic pipeline

The overall workflow and subject selection process are described in Fig. 1. Primary analyses were performed on paired microbiome-metabolome data sets on samples collected at approximately 6 weeks (*N* = 158 samples) and 12 months (*N* = 282 samples) of age (65 subjects have paired samples collected at both time points). In order to take advantage of the most samples from this study, each time point was analyzed separately with sensitivity analyses performed on sample pairs. As such, the sample size N will thereafter represent the number of samples found in each time point rather than the number of unique infants. After processing and filtering, we evaluated a final taxonomic dataset of 48 genera in 6 weeks samples and 72 genera in 12 months samples. Metabolomic profiles were represented as 208 unique untargeted spectral bins and a concentration-fitting method [26] was used to acquire specific relative concentrations of 36 targeted metabolites. These metabolites were chosen based on a literature search (Table S1) for compounds known to be associated with commensal gut microbes. Results presented here will primarily feature the targeted dataset, with accompanying figures and tables for the untargeted data set in the supplemental section. Figure 1 shows the overall workflow including the sample selection process. In summary, we performed three analyses: First, an overall concordance analysis using ordinations with ecological distances; second, a parametric multivariate correlation approach with a variable selection component to quantify the overall correlation and determine important factors that contribute to the overall microbiome-metabolite association; third, a predictive analysis approach to see if taxonomic abundance alone can accurately predict the concentrations of specific metabolites.

### Study population

Study subject characteristics are summarized in Table 1 separately for both subjects providing samples at 6-week of age (*n* = 158) and 12-months of age (*n* = 282). Characteristic of our population, most infants are White (97.5% among subjects contributing a 6-week sample; 95.4% among subjects contributing a 12-month sample), delivered vaginally (6 weeks samples: 72.2%; 12 months samples: 70.9%), and did not have any systemic antibiotic exposure during initial hospitalization following birth (6 weeks samples: 95.6%; 12 months samples 97.2%). The average birth weight was also similar across subjects irrespective of the sample time point, 3370 g (± 480) for infants contributing 6-week samples and 3430 g (±528) for infants contributing 12-month samples. Similarly, the average gestational age was 39.1 weeks (± 1.59) (6-week samples) and 39 weeks (± 1.7) (12-month samples). At the time of 6-week sample collection, 62% of infants had been exclusively breastfed while by the time of 12-month sample collection, 59.2% of infants received breast milk supplemented with formula, however a large minority (35.1%) remained exclusively breastfed. There were more male than female infants in the cohort (53.8% male among infants contributing a 6-week sample; 56.4% male among infants contributing a 12-month sample). Maternal smoking during pregnancy was rare (6-week samples: 7%; 12-month samples: 5%).

**Table 1:** Selected characteristics of subjects contributing samples at 6 weeks (*n* = 158) and at 12 months of age (*n* = 282)

	6 weeks (*n* = 158)	12 months (*n* = 282)
Birthweight (grams)		
Mean (Standard Deviation)	3370 (480)	3430 (528)
Median [Minimum, Maximum]	3430 [1910, 4710]	3450 [1320, 4660]
Missing	2 (1.3%)	4 (1.4%)
Sex		
Male	85 (53.8%)	159 (56.4%)
Female	73 (46.2%)	123 (43.6%)
Feeding Mode Until Time of Sample Collection		
Unknown	6 (3.8%)	7 (2.5%)
Exclusively breastfed	98 (62%)	99 (35.1%)
Exclusively formula fed	13 (8.2%)	9 (3.2%)
Mixed	41 (25.9%)	167 (59.2%)
Delivery Mode		
Vaginal	114 (72.2%)	200 (70.9%)
Cesarean	44 (27.8%)	82 (29.1%)
Gestational Age (Weeks)		
Mean (SD)	39.1 (1.59)	39.0 (1.70)
Median [Minimum, Maximum]	39.1 [33.4, 43.0]	39.1 [29.1, 42.0]
Post-delivery infant systemic antibiotic exposure		
No	151 (95.6%)	274 (97.2%)
Yes	7 (4.4%)	8 (2.8%)
Maternal smoking during pregnancy		
No	143 (90.5%)	262 (92.9%)
Yes	11 (7.0%)	14 (5.0%)
Missing	4 (2.5%)	6 (2.1%)
Infant Race		
Other	4 (2.5%)	13 (4.6%)
White	154 (97.5%)	269 (95.4%)

### Inter-omic sample distance comparison suggests overall concordance between data sets

**Fig. 2:**
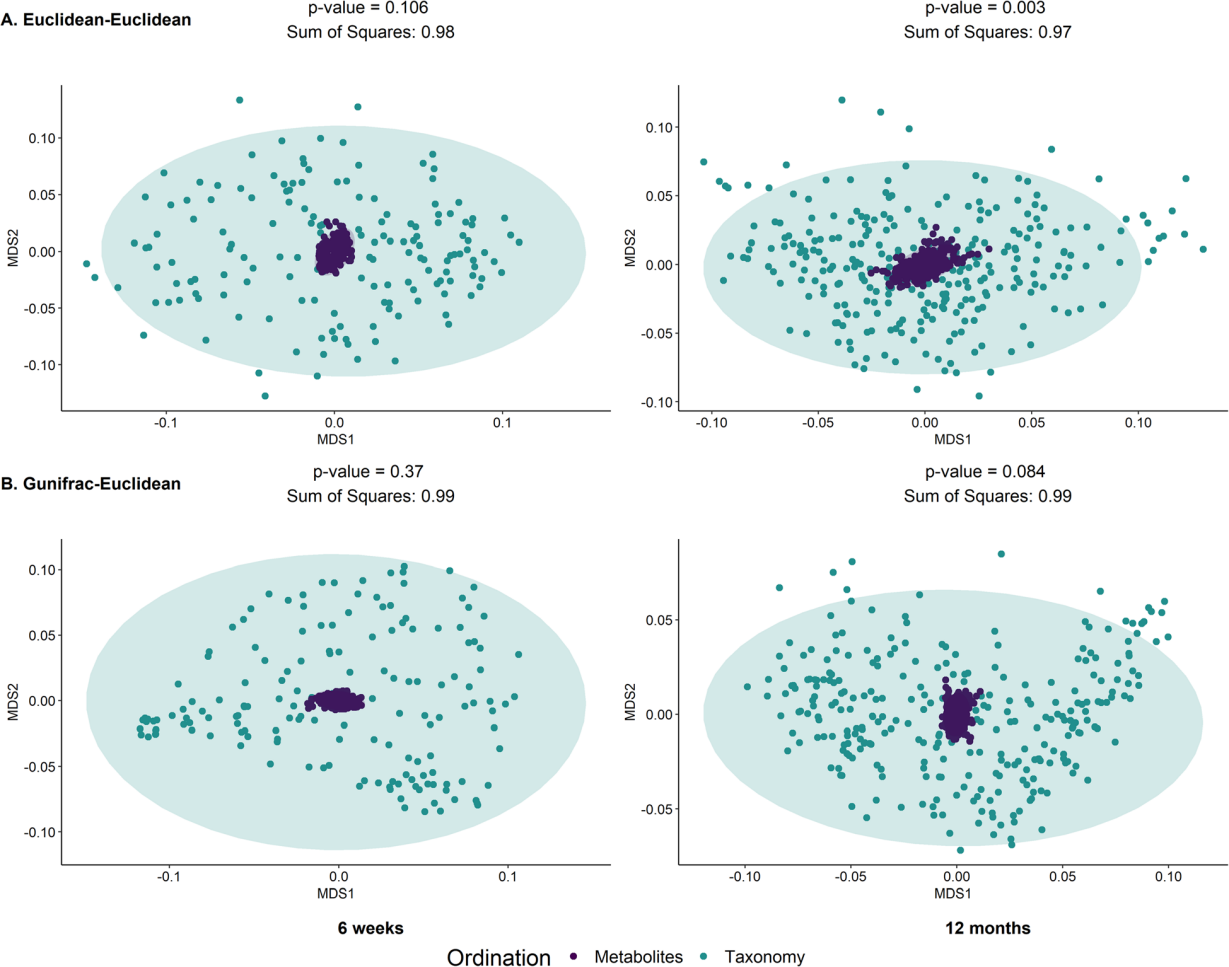
Inter-omics Procrustes biplots comparing PCoA ordinations of targeted metabolite profiles and taxonomic relative abundances for 6 weeks (left panels) (*n* = 158) and 12 months (right panels) (*n* = 262). Top panels present analyses based on ordinations from Euclidean distances of genus level abundances after centered log ratio transformation and Euclidean distances of log-transformed metabolite profiles. Bottom panel presents analyses based on gUniFrac distance of amplicon sequence variant (ASV) relative abundances and Euclidean distances of log-transformed metabolite profiles. There were significant associations between the microbiome and the metabolome (both targeted and untargeted) when utilizing Euclidean distances, however this association goes away when the gUniFrac distance was employed for the targeted metabolites only

Global concordance between the microbiome and the metabolome was observed across both time points and metabolomic data sets (Fig. 2A, Figure S1A) when analyzed using a symmetric Procrustes test with samples ordinated by Euclidean distances (Methods). It is noted that the p-value at 6 weeks for the targeted data set (*p* = 0.057) was only trending close to significant at the 0.05 level.

Since the Procrustes test was performed on principal coordinate (PCoA) ordinations of sample distances, the result is sensitive to the choice of dissimilarity metric. In addition to standard Euclidean distances, the generalized UniFrac (gUniFrac) metric was also leveraged to account for phylogeny in calculating differences between samples. With gUniFrac, the association was not significant at either time points for the targeted data set only (Fig. 2B), while the untargeted data set still maintained strong concordance (6 weeks samples – *p* = 0.001; 12 months samples – *p* = 0.006; Figure S1B).

### Sparse canonical correlation analyses reveal the core set of microbe-metabolite groups driving inter-omic relatedness

Given broad concordance between the gut microbiome and metabolome from sample distance analyses, we employed sparse canonical correlation analysis (SCCA) and pairwise Spearman rank correlation to ascertain the strength of association as well as to identify core microbes and metabolites driving this relationship (Methods).

The majority of taxa (65% of total genera at 6-weeks and 80% at 12-months) and metabolites (100% of total metabolites at 6-weeks and 80% at 12-months) were part of significant (FDR threshold 0.05) Spearman pairwise comparisons (Supplementary Note 1). This demonstrated a high level of congruence, where most microbes are involved in metabolic processes captured in the stool metabolome. This was also reflected in the significant multivariate correlation (permutation p. < 0.001). However, at 6 weeks (correlation: 0.606 [0.61–0.73]), the degree of concordance was slightly higher than at 12 months (correlation: 0.52 [0.431–0.646]) but this difference was not significant due to overlapping confidence intervals. The canonical correlation was overall slightly higher in the untargeted data set (6 weeks: 0.636 [0.621–0.733]; 12 months: 0.49 [0.475–0.702]), however the difference between time points was similar (Figure S2, Supplemental Note 2).

**Fig. 3:**
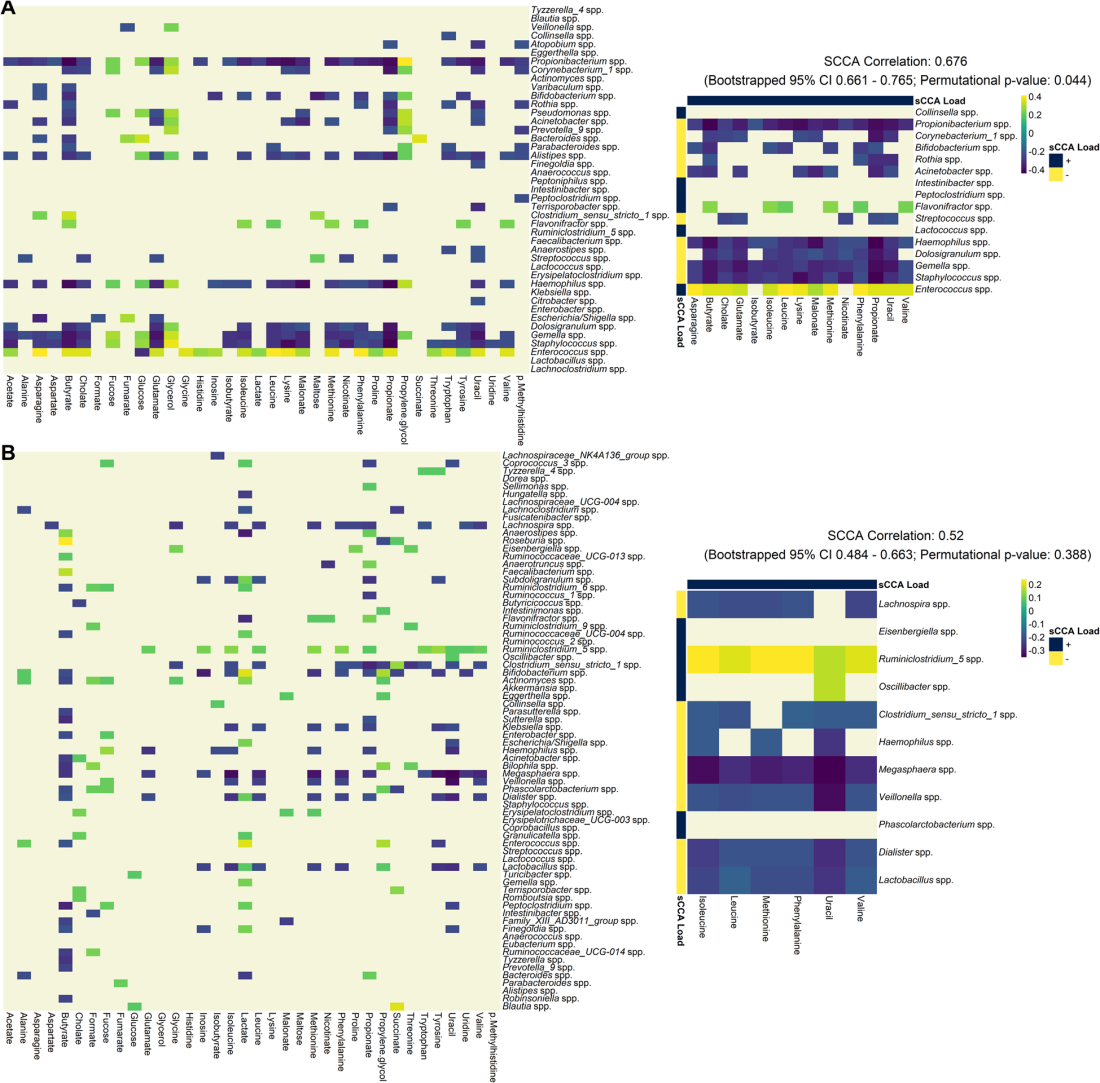
Pairwise Spearman correlation of concentration-fitted metabolites and genus-level taxonomic abundances for 6-weeks (panel A, *N* = 158) and 12-months (panel B, *N* = 282) infants. Left panel displays the overall correlation pattern, where non-significant correlations are not colored (false discovery rate (FDR) controlled q-value < 0.05). Right panel displays the same heatmap restricted to taxa and metabolites selected by the sparse CCA procedure. Additionally, correlation coefficient of the first sCCA variate pair, bootstrapped 95% confidence interval and permutation *p*-value are also reported. Significant microbiome-metabolome correlation was observed at both time points, however no significant difference was found between the time points

Using SCCA, we identified a core set of microbes and metabolites that are major contributors to the multivariate correlation (Fig. 3, right panels; Supplementary Notes 2). Selected microbes (in both the targeted and untargeted data set) belonged to the Firmicutes, Actinobacteria and Proteobacteria phyla, as those are the most commonly found phyla in the infant gut [25, 27]. However, previously established dominant genera such as *Bifidobacterium*, *Bacteroides* and *Lactobacillus* were not consistently selected across both time points. In the targeted data set *Bifidobacterium* was selected only at 6 weeks and *Lactobacillus* was only selected at 12 months. Most notably, more microbes were selected at 12 months compared to 6 weeks in the targeted data set, however in the untargeted data set this pattern was reversed (Figure S2, right panels). The majority of the selected metabolites in the targeted data set were amino acids (Supplementary Table 1), with some short chain fatty acids (SCFAs) selected at the 6-week time point.

### Microbial community structure is weakly predictive of stool metabolite relative concentrations

In order to determine how well the fecal metabolome acts as a functional representation of the gut microbiome, we fitted metabolite-specific prediction models based on taxonomic profiles. Chosen models include random forest (RF), elastic net (EN), support vector machines with radial basis kernel (SVM-RBF) and sparse partial least squares (SPLS), all of which had previously been shown to work well with microbiome-associated learning tasks [28]. Evaluation was based on predicted R-squared (R^2^) and Spearman correlation coefficient (SCC) as measured using 100 repeats of 5-fold nested cross validation (Methods).

**Fig. 4:**
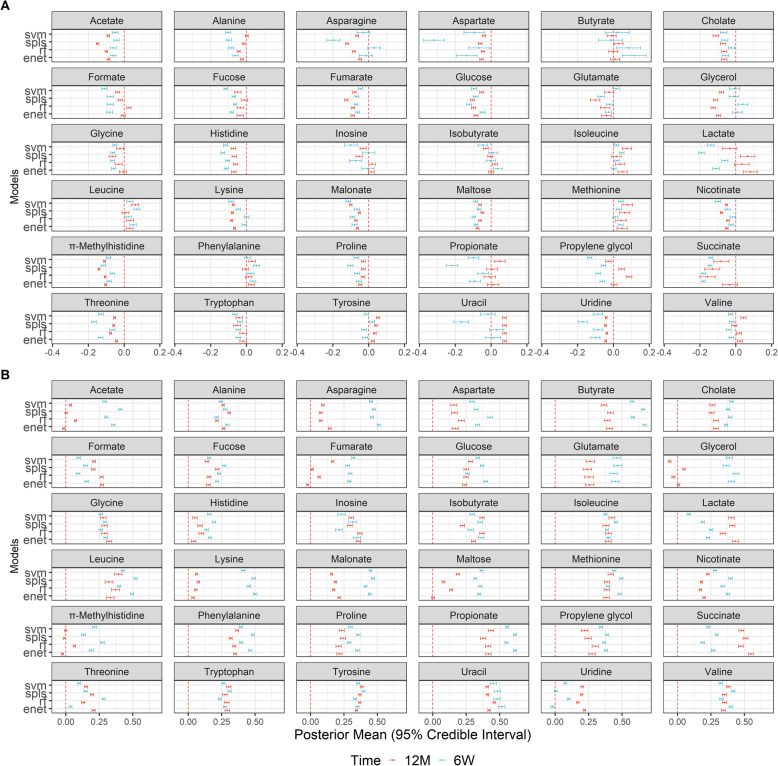
Forest plots of each prediction performance metric (R-squared – Panel **A**, Spearman correlation – Panel **B**) for each time point (6 weeks (*n* = 158), 12 months (*n* = 282)) across all 36 metabolites and 4 machine learning models. 95% credible interval and predictive posterior means were generated using Bayesian modelling of the evaluation statistic (Methods) after 100 repeats of 5-fold nested cross validation. Red vertical lines indicate a value of 0 for the evaluation metric (equivalent to null model). Metabolites were classified as predictable if the null value did not lie within the estimated 95% credible interval. For most metabolites, predictive performance was not significantly better than null models

Predictive performance was more dependent on the metabolite being predicted than by choice of model (Fig. 4, Supplementary Notes 3, Supplementary Files 1). Looking at predictive R^2^ (Fig. 4 panel A), the average posterior mean performance across all models and metabolites was negative for both time points (− 5.6% at 6 weeks; − 3.07% at 12 months), which indicated that for most prediction tasks the fitted model was less predictive than a naïve, intercept only model. At 6 weeks 22.2% of metabolites had models that perform significantly better than the null (lower bound of 95% credible interval larger than 0) while at 12 months 38.9% of metabolites fit the classification. However, even among such metabolites, the maximum R^2^ is only 11.8% at 6 weeks and 8.7% at 12 months. Conversely, SCC values were higher in comparison (cross-metabolite avg.: 0.339 at 6 weeks and 0.249 at 12 months) (Fig. 4 panel B, Supplementary Notes 3). At 6 weeks, 83% of metabolites were significantly more performant than the null, while at 12 months all metabolites were selected. Using a more stringent cutoff as used by Mallick et al. [29], the majority of metabolites at 6 weeks (69.4% of total metabolites) still remained as well predicted while conversely at 12 months only 38.9% (of total metabolites) were predictable.

Results from the untargeted analysis showed higher performance values for both evaluation metrics (Supplementary Note 3). Specifically, 56.7% of metabolites bins at 6 weeks and 42.7% of bins at 12 months had R^2^ values significantly higher than 0. However, under SCC, while 57% of metabolite bins at 6 weeks had SCC values significantly larger than 0.3 cutoff, only 28.8% of metabolite bins at 12 months fit this criterion. Despite better performance, the overall average values were still low, suggesting that across the entire metabolome few metabolites were highly predictable.

**Fig. 5:**
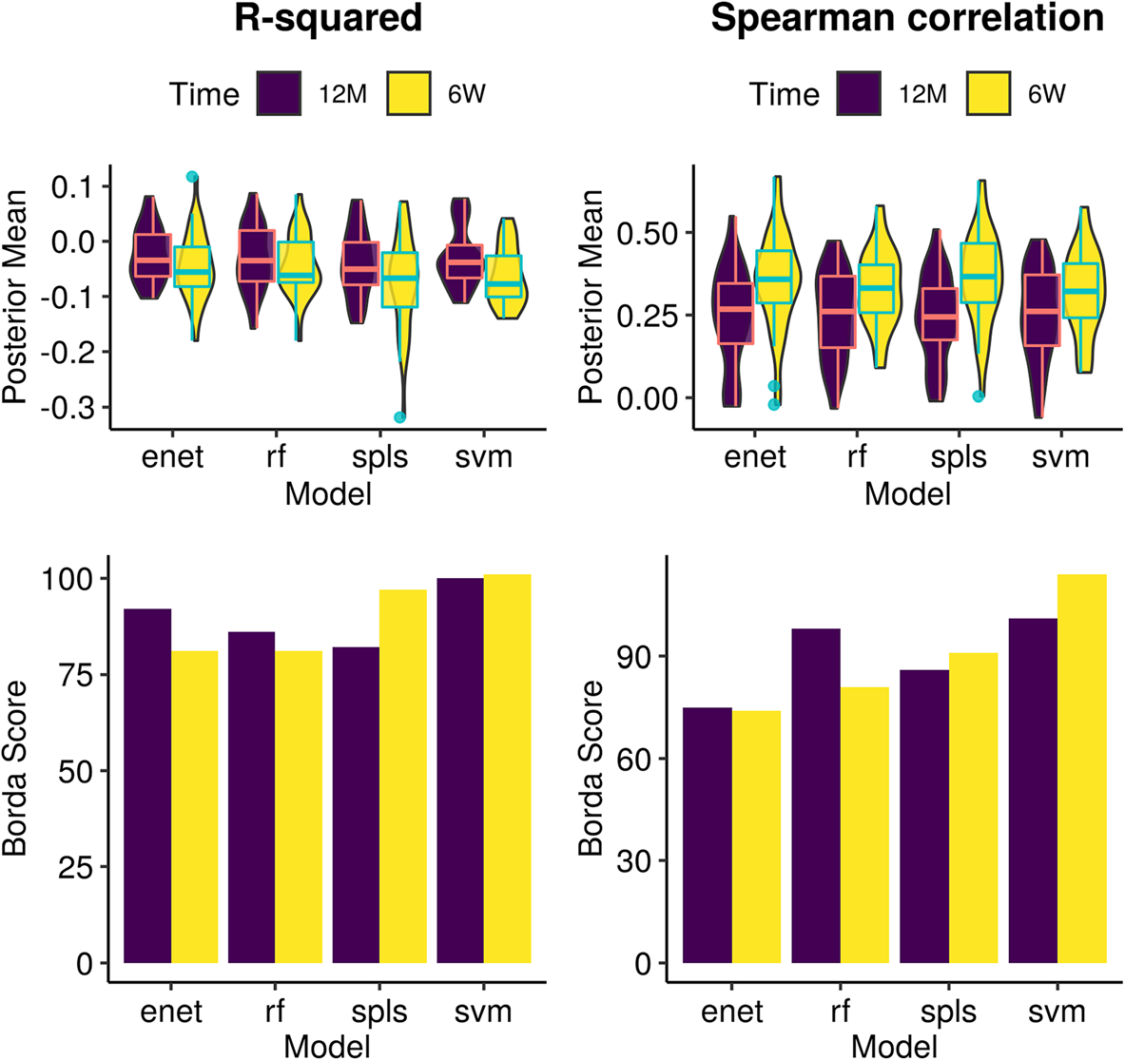
Comparative analysis predictive model performance across all metabolites in the targeted dataset for both 6-weeks (*n* = 158) and 12-months (*n* = 282) time points. Top panel shows superimposed boxplots and violin plots of the distribution of predictive posterior mean for each evaluation metric across all 36 metabolites. Bottom panels show aggregated model rankings for all metabolites using R-squared (left) and Spearman correlation (right) using Borda scores (Methods). Higher scores indicate that a model was consistently selected as a better performing. Relatively similar Borda scores and cross-metabolite average predictive performances indicate that no model was clearly the most performant. However, support vector machines (with radial basis function kernel) was highest scoring model

Despite weak predictive performance values, we were still interested in determining a model that stands out as the most appropriate for our prediction task. Aggregating performance across metabolites stratified by model for both evaluation metrics (Fig. 5, top panel), it can be observed that the average performances were similar (Supplementary Notes 3), for which no semi-targeted analyses performed better on average than the naive model under R^2^. This is further illustrated when model performance was aggregated by rank using Borda scores (Fig. 5, bottom panel). A higher score indicated that a model was selected as the top choice more times than others, where an even score distribution across models corroborated the suggestion that no model was best across all prediction tasks. That said, SVM-RBF seemed to be the highest scoring model, particularly for the 6-week time point. The untargeted analysis also found similar results (Figure S3).

### Sensitivity analyses

We performed both Procrustes and correlation analyses on a data set restricted to the 65 subjects with paired samples collected at both time points (6 weeks and 12 months). Each time point was analyzed separately similar to our main analysis. In the targeted data set, significant Procrustes concordance was observed at 12 months (*p*-value = 0.003) but not at 6 weeks (*p*-value = 0.106). This association was no longer significant when considering taxonomic ordination using the gUniFrac distance metric (6 weeks). Surprisingly, in the untargeted data set, no association was observed across both time points and choice of distance metric (Figure S5, S6). In the canonical correlation analyses, significance was only observed in the targeted data set at 6 weeks only (6 weeks: permutation p-value = 0.044; 12 months: permutation p-value = 0.388). Even though most correlations were not significantly different from the permuted null, the canonical correlation coefficient is higher at 6 weeks compared to 12 months in both the targeted (6 weeks: 0.676 [0.661–0.765]; 12 months: 0.52 [0.484–0.663]), and untargeted (6 weeks: 0.703 [0.685–0.788]; 12 months: 0.444 [0.52–0.705]) data sets (Figure S7, S8).

Furthermore, to ascertain the uncertainty of model choice, we evaluated all selected modelling approaches with simulated data sets based on bootstrapped resampling of taxonomic relative abundances (Figure S4). For the first simulation scenario, models were assessed against generated metabolite concentrations under different degrees of model saturation (number of taxa associated with the outcome) and association strength (signal to noise ratio). As expected, model performance asymptotically reached perfect prediction with increasing signal strength and model saturation, which demonstrated that prediction models were able to capture predictive associations should they arise even in sparse microbiome data sets. Most notably, simulation performance differed more by signal-to-noise ratio than model saturation, which indicated that the strength of association plays a larger role in the observed weak predictive performance than the number of taxa involved. Surprisingly, we obtained very similar results to our real data values under our lowest simulation setting (model saturation = 5%; signal-to-noise ratio 0.5). As such, it can be suggested that the lack of predictability is due to weak coupling rather than model choice.

## Discussion

In this study, we provide a descriptive and hypothesis generating analysis of the relationship between fecal microbial taxonomic abundances and metabolite concentrations with multi-omic profiling via paired targeted sequencing of the 16S rRNA gene and H^1^ NMR metabolomics at multiple time points. Ecological, statistical and machine learning approaches were applied to provide a multi-faceted view of this association. To our knowledge, this study is one of the few comprehensive investigations addressing the microbiome/metabolome interface in a large general population cohort of infants.

### The microbiome is significantly correlated but weakly predictive of the metabolome

Overall global concordance was found from three independent methods (Procrustes analysis, SCCA and univariate Spearman correlation), consistent with previous studies on both infant [19, 24] and adult populations [17, 30]. This overall effect was found at both time points, suggesting there coupling exists throughout infancy despite high levels of both inter- and intra-individual variability in taxonomic compositions [27].

Although our analyses demonstrated significant multivariate and univariate correlation between the microbiome and the metabolome, most metabolites were not predictable when evaluated across multiple machine learning models. Even among the small number of metabolites that are significantly predictable compared to the null, the maximum performance values were still low for both the untargeted and targeted analyses. When compared to a recent study performing metabolite predictions from taxonomic abundances using an adult cohort [29], both the number of well-predicted metabolites and the average performance values were much lower, even when using similar evaluation criterion and cut offs. It is unlikely that model choice was driving the lack of predictability, since all chosen methods had been shown to be suited for microbiome-associated prediction tasks [28, 31] as well as covering both linear and non-linear associations. This is further evidenced in our sensitivity analyses, where non-parametric simulations demonstrated that low predictability across both evaluation metrics was driven by low signal-to-noise ratio rather than model choice or number of taxa driving the association.

These results can be attributed to the limitations of our study design. We utilized partial 16S rRNA sequencing instead of whole genome shotgun sequencing. This limits our taxonomic resolution to the Genus level for most of the analysis [32]. Since bacterial functions relevant to human metabolism are likely to be strain specific [33, 34], we hypothesized that aggregating to Genus level might dilute the direct effects, where different strains within the same Genus might have opposite impacts on the abundance of a certain metabolite. This would result in a lack of predictability as the same feature would contain elements that both increase and decrease the values of the outcome of interest.

However, we can potentially attribute overall performance to other ecological processes. A likely candidate is functional redundancy, an aspect ubiquitous in microbial communities [35], plays an important role in this weak coupling. Functional redundancy is the ecological phenomena that multiple species representing a spectra of taxonomic groups can perform similar roles [35, 36], and is usually a marker for ecosystem resilience [37]. Under this paradigm, the loss of a certain metabolite producing taxon would not impact the abundance of that metabolite as other taxa in the community can complement the functional role, complicating taxa to metabolite predictions. This is evidenced in the Procrustes analysis where inter-omic associations are no longer significant in Procrustes analyses when phylogenetic relatedness was adjusted using the gUniFrac distance metric. Since gUniFrac adjusts for phylogeny by weighting the differences in proportions of each taxa across two samples by the branch length from constructed evolutionary trees [38], the absence of an association suggests that samples with similar metabolic profiles might be numerically comparable (cluster together under Euclidean distances) but with evolutionarily divergent taxonomic compositions. This is further supported by our supplementary PICRUSt2 analyses, where we found for most pathways no single genera dominate functional contribution (Figure S10). Functional redundancy is also consistent with previous research in human associated microbiomes [39].

### Taxa and metabolites selected to be core to the microbiome-metabolome correlation reveal the importance of amino acid metabolism

Taxa and metabolites with non-zero loading coefficients in SCCA analyses were identified as factors driving this overall correlation. The SCCA procedure utilized a L_1_-penalized matrix decomposition of the cross-product matrix akin to a LASSO regression problem [40], which means that variables were selected based on their importance to the overall covariance between taxa and metabolite abundances.

At 6 weeks, two short chain fatty acids (SCFAs), butyrate and propionate, were selected as core to the microbiome-metabolome interface. SCFAs (which includes compounds such as isobutyrate, and acetate) are important metabolites obtained primarily from colonic microbial fermentation of carbohydrates that escape digestion in the small intestines [41]. Butyrate is an energy source for colonocytes [42] as well as participating in the maintenance of the gut epithelial barrier through mucin production [43]. Similarly, propionate is part of the gluconeogenesis pathway in liver hepatocyte cells, which is core to lipid and energy metabolism in liver [44]. Most importantly, SCFAs participate in immune programming in early life, where the reduction in SCFA producing bacteria is associated with inflammatory bowel disease [45, 46].

SCFA production in early life is linked to the *Bifidobacterium* and *Bacteroides* catabolism of human milk oligosaccharides (HMO) [47–49], which explains the selection of the *Bifidobacterium* genera at 6 weeks where infants are exclusively on a milk-based diet. This is further supported in our supplementary PICRUSt2 analysis, where predicted pathways whose abundance significantly correlate with butyrate concentrations were those associated with breakdown of sugars into butanoate (Figure S9). The genera breakdown of those functions features prominently *Bacteroides, Bifidobacterium, Lachnoclostridium, Flavonifractor,* and *Clostridium* sensu stricto *1* genera (Figure S10). This demonstrates that at 6 weeks, infant microbiome-metabolome interaction is primarily concerned with breakdown complex sugars into SCFAs, cementing it’s functional role in microbiome development [50].

Surprisingly, the selected *Bifidobacterium* genus is negatively correlated with butyrate abundance. We hypothesized that this might be due the complex cross-feeding relationship that exist between *Bifidobacterium* and butyrate-producing taxa [51]. On one hand, some *Bifidobacterium* species can be completely commensal, producing secondary metabolites such as acetate that assist in the growth of butyrate producing species. On the other hand, other *Bifidobacterium* strains such as *B. longum* LMG 11047 and *B. adolescentis* can compete for the same substrates as butyrate producing species [52]. The selection of the negative association between *Bifidobacterium* and butyrate suggests that butyrate-suppressing *Bifidobacterium* strains might be more important in our infant samples.

However, the most selected metabolites in SCCA analyses are amino acids (7 out of 10 metabolites selected at 6 weeks were amino acids). Prior studies have shown that the microbiota participate in regulating host amino acid homeostasis by acting as both producers and utilizers [15]. The most common amino acid fermenters in the human gut include those from the *Clostridia* class [53]. Our results further support this as most selected microbes with positive correlation with amino acids are of the *Eisenbergniella, Flavonifractor, Ruminococcaceae UCG-004, Oscillibacter* and *Ruminiclostridium* genera under *Clostridia*. This is further seen in our supplemental PICRUSt2 analyses, where predicted abundance of isoleucine and methionine biosynthesis pathways are significantly correlated with observed concentrations (Figure S9).

Aside from being fermenters, microbes can also either directly utilize amino acids and incorporate them into protein synthesis, or catabolize them as an energy source, producing secondary metabolites. Even though the process of amino acid catabolism for energy alone is not energetically efficient [10], it produces secondary metabolites such as the aforementioned SCFAs, which are important molecules in the metabolic interactions between the microbiota and the host. However, amongst selected microbes whose abundance are negatively correlated with amino acid concentrations (hence, suggestive of catabolism), we do not observe corresponding positive correlation with selected SCFAs. We hypothesized that this might be due to the fact that bacterial concentrations are higher in distal parts of the intestine [9, 15] where nutrient availability is low. This lack of available carbohydrates might incentivize microbes to conserve energy by directly incorporating free amino acids rather than metabolizing them. On the other hand, prior studies suggested that microbial amino acid catabolism is compartment specific and occurs in more proximal regions [53, 54]. However, our study design is limited to cross-sectional metabolomic profiling, which limits the possibility of detecting SCFAs that are rapidly produced and absorbed.

### The microbiome is more tightly coupled with the metabolome in early infancy

Results suggest some level of significant difference in microbiome-metabolome coupling across development. Canonical correlation, while not significantly different, were lower at 12 months than at 6 weeks, suggesting a time-varying effect. When looking at predictability, we observed a higher number of well predicted metabolites at 6 weeks compared to 12 months. Among those selected as well predicted metabolites, the average performance values (both R^2^ and SCC) where higher. This is also replicated in the global untargeted data set. Furthermore, in our supplementary PICRUSt2 analyses, there exists a higher number of significantly correlated predicted pathway abundance to observed metabolite concentrations (Figure S11), indicating increased metabolic coupling between the microbiome and the metabolome at 6 weeks compared to at 12 months.

There are various factors that can contribute to the difference in microbiome-metabolome coupling between infants at 6 weeks and 12 months. First, there exists substantive differences in dietary patterns for those included in our analysis. The majority of infants at 6 weeks (62%) were exclusively breastfed, while that number is markedly less (35%) at 12 months, where infants are also consuming complimentary solid family foods. This transition in diet to solid foods have been shown to induce a change in the gut microbiome composition and diversity due to increased amounts of fiber and protein [55, 56], which might favor certain microbes over others. Such changes in diet, particularly the cessation of breastmilk intake, also contributed towards the development of infant gut microbiomes towards a more “adult like” state [27, 55]. We hypothesized that earlier in life when infants are only consuming a limited type of food (predominantly breast milk or formula), the microbiome participates more actively in host-microbiome co-metabolic activity as infants are more reliant on microbes to breakdown complex nutrients [57]. Conversely, at 1 year of age where the microbiome has matured, this relationship is not as strongly coupled as a larger share of the metabolome comes from host-produced metabolites.

However, as analyses were conducted within each timepoint independently with little subject overlap, further investigations are required to make more conclusive statements about the potential time-varying effect of microbiome-metabolome coupling. Particularly, aside from differences in diet, factors such as differences in antibiotic exposure [58] and maternal covariates [59] might result in differences between time points. In future studies we hope to examine this factor using samples across multiple time points for the same infants.

### Limitations

This study has various limitations. First, we utilized partial 16S rRNA gene sequencing instead of shotgun whole genome sequencing, which limits our taxonomic resolution to the genus level for most of the analysis [33]. We hypothesized this lack of resolution contribute to overall lack of predictability, as well as limiting the interpretability of variables selected by the SCCA process as species and strain level differences can result in completely separate metabolic contributions [34]. For example, we cannot disentangle the different *Bifidobacterium* strains that might compete with butyrate producing taxa and generating the negative correlation between measured SCFAs and *Bifidobacterium* abundance.

Second, our cohort includes only infants from the NHBCS, a population-based cohort reflecting mostly rural and White demographics of northern New England in the United States. While this increases confidence in the internal validity of our study, this homogeneity in race and geography limits the generalizability of our results to other populations.

Third, our study is a cross-sectional survey of microbiome-metabolome relationships at two different time points. This means that we cannot capture associations relating to metabolites that are highly produced and consumed. This means that the metabolites selected might not be representative of the intricate relationship between the microbiome and the metabolome. This interpretation is further limited by the lack of annotation for our untargeted metabolite bins, which cannot be compensated by the small number of metabolites selected for the targeted analyses.

Finally, each time point was analyzed independently with only 65 subjects with samples in both time points. As such, this limits the ability to explore the differences in coupling across the first year of life.

## Conclusion

In conclusion, we conducted one of the first large-scale multi-omics analysis of the microbiome-metabolome relationship using samples from a large birth cohort study at 2 time points (6 weeks and 12 months). Although we found global concordance between the microbiome and the metabolome, the inter-omic concordance is weak, where bacterial abundances at the genus level cannot accurately predict metabolite concentrations. We hypothesized that this might be due to functionally relevant diversity at the strain level, as well as the impact of functional redundancy on the contribution of each microbe to metabolite abundances. Additionally, we were able to identify metabolites and microbes driving the overall correlation. Results pointed to support the importance of SCFA metabolism particularly at 6 weeks, as well as the role of amino acid metabolism, either as a source of SCFA and energy in the absence of carbohydrates, or as a general mechanism for microbes to save energy as they incorporate amino acids around their environment. Finally, our analysis suggests preliminary evidence that the degree of microbiome-metabolome coupling changes across time, being much more integrated at 6 weeks compared to 1 year.

We conclude that although the metabolome is a functional output of the microbiome, there exists massive challenges in being able to trace specific microbial contributions to host-microbe metabolism due to the complexity of factors such as functional redundancy and strain level variability. As such, we recommend studies to profile both the microbiome and the metabolome, as aspects of microbial metabolic contributions cannot be found solely through one omic data set. This is particularly important in settings where it is important to have a mechanistic understanding of the role of microbes such as developing of microbiome therapies [60].

## Methods

### Study population

Subjects for this study were from the New Hampshire Birth Cohort Study (NHBCS) who provided infant stool samples at 6-weeks and 12-months after birth. These two timepoints are chosen as each correspond to routine maternal postpartum visit, allowing sample collection with minimal participant burden. Furthermore, at both time points, infant feeding patterns are comparatively more well established. As described in previous studies [25, 59], NHBCS is a prospective study of mother-infant dyads in New Hampshire, USA. Participants eligible are pregnant women between the ages of 18 and 45 years old, currently receiving routine prenatal care at one of the study clinics, consuming water out of a private well with no intention to move prior to delivery. The Center for the Protection of Human Subjects at Dartmouth provided institutional review board approval. All methods were carried out in accordance with guidelines. Written informed consent was obtained for participation from all subjects for themselves and their children. Comprehensive sociodemographic, exposure and outcome data such as infant feeding method, delivery mode, maternal smoking status, etc. were collected for all participants through surveys, medical records and telephone interviews conducted during pregnancy, about 6 weeks postpartum, and updated every 4 months up until first year of age and every 6 months thereafter.

### Collection of infant stool samples

Infant stool samples were collected at 6-weeks and 12-months. Stool samples were provided in diapers and stored by subjects in their home freezer (− 20 °C) until they were able to return it to the study site. Stool was thawed at 4 °C so that it could be aliquoted into cryotubes. Stools collected for 16S rRNA gene sequencing were aliquoted (range 350–850 mg) into 3 ml RNAlater and homogenized before storing at − 80 °C. Stools collected for metabolomic analysis were aliquoted (1–2 g) into 15 ml centrifuge tubes before storing at − 80 °C.

### Taxonomic profiling using 16S rRNA targeted gene sequencing

RNAlater stool samples were thawed and DNA was extracted using the Zymo Fecal DNA extraction kit (Cat# D6010, Zymo Research, Irvine, CA), according to the manufacturer’s instructions. For each sample extraction, 400ul RNAlater stool slurry (50–100 mg of stool) was used to isolate DNA. Extractions were performed in batches of multiple samples and included a composite RNAlater stool positive control and a RNAlater negative control. Lysis was performed using 750ul Lysis Buffer in ZR BashingBead™ Lysis Tubes (0.5 mm beads), mixed and then shaken on a Disruptor Genie for 6 min. Eluted DNA was quantified on a Qubit™ fluorometer using the Qubit™ dsDNA BR Assay. Average coefficient of variation of DNA yields (ng/ul) for composite RNAlater stool positive controls was 28%. No DNA was ever detectable in negative control elutions. Concentrations of DNA samples used for 16S rRNA gene sequencing range from 1 ng/ul to 25 ng/ul.

The V4-V5 hypervariable region of bacterial 16S rRNA gene was sequenced at Marine Biological Laboratory in Woods Hole, MA, using standard Illumina MiSeq amplicon approach (paired end sequenced between 518F and 926R) [61, 62]. As described previously [25, 59], 16S rDNA V4-V5 amplicons were generated from purified genomic DNA samples using fusion primers. The use of forward primers containing one of eight five-nucleotide barcodes between the Illumina-specific bridge and sequencing primer regions and the 16S-specific region and a single reverse primer containing 1 of 12 Illumina indices enables 96 samples per lane multiplexing. Amplifications were done in triplicate with one negative control for internal quality control at MBL. We used qPCR (Kapa Biosystems) to quantify the amplicon pool, and one Illumina MiSeq 500 cycle paired end run to sequence each pool of 96 libraries. We demultiplex and divided datasets using Illumina MiSeq reporter and a custom Python script. Demultiplexed reads derived from Illumina sequencing were denoised and quality filtered using DADA2 (v. 1.12.1) [63] in R (v. 3.6.1) [64]. Illumina adapter sequences were removed prior using cutadapt (v. 1.18). We utilized DADA2’s *filterAndTrim* function to remove reads either containing a quality score of 2 or lower (*minQ = 2*) or with expected errors [65] of 2 (*maxEE = c (2,2)*) or higher. Post filtering, we obtained an average of 119,800 reads per sample for 6-week samples and 120,480 reads per samples for 12-month samples. On average, we 74.7% of reads were kept for 6-week samples and 76.3% of reads were kept for 12-month samples. We then use the RDP classifier implemented natively in the DADA2 R package with SILVA database (v. 128) to profile the taxonomy of identified amplicon sequence variants (ASVs).

### Functional profiling using untargeted and targeted ^1^H NMR metabolomics

^1^H NMR metabolomics was performed in collaboration with the NIH Eastern Regional Comprehensive Metabolomics Resource Core (RCMRC) at UNC Chapel Hill. De-identified stool aliquots were shipped on dry ice and immediately stored at − 80 °C for metabolomics analysis. Samples were thawed and ~ 150 mg of stool samples were transferred to MagNA Lyser tubes after recording the weight. Samples were then homogenized with 50% acetonitrile in water by using the Omni Bead Disruptor (Omni International, GA, USA). Homogenized samples were centrifuged at 16000 rcf and the supernatant was separated into another tube. An aliquot (1000 uL, 100 mg equivalent of fecal mass) was transferred into an Eppendorf tube and lyophilized overnight. The dried extract was reconstituted in 700 uL of NMR master mix (containing 0.2 M phosphate buffer, 0.5 mM DSS-d6 (internal standard), and 0.2% sodium azide (preventing bacterial growth)), vortexed on a multi tube vortexer at speed 5 for 2 min and centrifuged at 16000 rcf for 5 min. A 600 μl aliquot of the supernatant was transferred into pre-labeled 5 mm NMR tubes. Additionally, study pooled quality control (QC) samples (created from randomly selected study samples) and batch pooled QC samples were generated from supernatants of study samples and aliquots of supernatants were dried and reconstituted similar to study samples described above and used for QC purposes.

^1^H NMR spectra of feces extracts were acquired on a Bruker 700 MHz NMR spectrometer using a 5 mm cryogenically cooled ATMA inverse probe and ambient temperature of 25 C. A 1D NOESY presaturation pulse sequence (noesygppr1d [66, 67], [recycle delay, RD]-90°-t1–90°-tm-90°-acquire free induction decay (FID)]) was used for data acquisition. For each sample, 64 transients were collected into 64 k data points using a spectral width of 12.02 ppm, 2 s relaxation delay, 10 ms mixing time, and an acquisition time of 3.899 s per FID. The water resonance was suppressed using resonance irradiation during the relaxation delay and mixing time. NMR spectra were processed using TopSpin 3.5 software (Bruker-Biospin, Germany). Spectra were zero filled, and Fourier transformed after exponential multiplication with line broadening factor of 0.5. Quality control measures included review of each NMR spectrum for line shape and width, phase and baseline of spectra, and tight clustering of QC samples in Principal Component Analysis [68]. NMR bin data (0.49–9.0 ppm) were generated (untargeted data) excluding water (4.73–4.85 ppm) using intelligent bucket integration of 0.04 ppm bucket width with 50% looseness using ACD Spectrus Processor (ACD Labs Inc., Toronto, Canada). The integrals of each bin were normalized to the total spectral intensity of each spectrum and transferred to analysis software. This resulted in a collection of spectral bins with bin-specific relative abundances, which will be called the untargeted data. In addition, relative concentration of library-matched metabolites (selected from the literature implicated to be important in host-microbe relationships - Table S1) was determined by using Chenomx NMR Suite 8.4 Professional software [26]. This data set will be called the targeted data set.

### Software and tools

All analyses were performed using the R programming language (v. 3.6.3) [64] and associated packages. All data wrangling steps were performed using *phyloseq* [69], *plyr* and *tidyverse* packages [70], as well as the *compositions* package [71] for log-ratio transformations. All figures were generated using the *ggplot2* [72], *cowplot* [73]*, viridis* [74] and *pheatmap* [75] packages. Additionally, the *tidymodels* [76] suite of packages was utilized to assist in all modelling tasks. Specific packages used for modelling will be enumerated below. All scripts as well as intermediary analysis objects are available on GitHub with all dependencies and their versions (https://github.com/qpmnguyen/infant_metabolome_microbiome).

### Data transformation and normalization

For microbiome data, we retained all ASVs present in at least 10% of samples [29] and added one pseudocount to all cells [77]. We then subsequently aggregated all ASVs to the genus taxonomic level [28] and converted data to relative proportions using total read counts by sample to account for differential sequencing depth. We further filtered out taxa with mean relative proportion < 0.005% [78]. This filtration step resulted in 46 genera for 6-week samples and 72 genera for 12-month samples. To address the compositional problem induced by a sum to one constraint, we apply the centered log ratio transformation (CLR), which is often used to remove such constraints in microbiome data sets [79]. The CLR transformation is favored compared to other statistically equivalent log-ratio transformations due to its scale invariant property and ease of interpretation [80].

For metabolomic data sets, we employed different transformations to approximate homoscedasticity depending on the data type (targeted vs untargeted). For targeted metabolites, we performed a *log*(*x* + 1) transformation while for untargeted metabolites we utilized the arcsine square root transformation which has been previously used for transforming composition metabolomics data sets [29].

### Distance matrix analyses

Principal coordinates analysis (PCoA) was performed using the *pcoa* function from the *ape* package in R [81] with sample distance matrices. The PCoA procedure seeks to represent high dimensional multivariate data sets in lower dimensions through eigen decomposition of the doubly centered distance matrix. PCoA allows the usage of non-Euclidean distances between samples such as ecological indices, which makes it a preferable method for sample ordination compared to principal component analysis (PCA).

We constructed Euclidean distance matrices for both metabolic and taxonomic profiles post data transformation described in the previous section. Additionally, gUniFrac distances (alpha = 0.5) [62] were considered for taxonomic data using the implementation provided in the package *MiSPU* [63]. gUniFrac requires a phylogenetic tree, of which an approximate maximum likelihood phylogenetic tree was constructed with representative ASV sequences using FastTree (v 2.1) [64]. Multiple sequence alignment was performed using the *AlignSeqs* function from the *DECIPHER* package in R [65] and trees were midpoint rooted using *phytools* [66]*.* Since multiple sequence alignment is not conserved under filtering and aggregation of ASVs, gUniFrac distance calculations were performed with pre-filtered ASV-level abundances normalized to relative abundances.

The first two axes of constructed ordinations were then compared using a symmetric Procrustes procedure implemented in the *protest* function in the *vegan* package [67]. Procrustes superimposes two ordinations by translating and rotating the coordinates, which preserves the general structure of the data. This method performs a superimposition fit between two data sets minimizing the sum-of-squared differences (m^2^), which describes the degree of concordance between the two configurations normalized to unit variance. Significance is obtained by testing against the permuted null using a permutation test. This method was shown to have more power while also limiting type I error compared to the traditional Mantel test in ecological analysis [82]. Significance was determined using a permutation test on the sum of squared differences with 999 permutations [68].

### Sparse canonical correlation and spearman correlation analyses

Sparse canonical correlation analysis (sCCA) was performed to identify strongly associated metabolite-microbe groups. sCCA seeks to find linear combinations of variables from each dataset that maximizes the correlation with each other while simultaneously thresholding variable specific weights to induce sparsity and performing variable selection. The correlation coefficient in the first canonical variate quantifies the overall degree of multivariate associations. As such, sCCA is a popular method in integrating multi-omics datasets with the ability to select more biologically relevant sets of features compared to traditional ecological methods such as co-inertia analysis [83]. In this study, we use the sCCA implementation in the package *PMA* in R [40] which uses a novel penalized matrix decomposition procedure to achieve sparsity [84]. We tune hyperparameters using a permutation approach in the *CCA.permute* function (nperms = 50) prior to fitting the final model. We obtain the correlation coefficients as a measure of overall correlation between the two data sets and calculated a bootstrapped 95% confidence interval (nboot = 5000) as well as performing a permutation test (nperm = 1000) at the 0.05 significance level. In order to keep the structure of the data set across different permutations, we use the function *randomizeMatrix* from the package *picante* in R [85] using the *richness* null model, which randomizes community abundances within samples to maintain sample species richness.

Pairwise Spearman correlations were also performed using the *cor* function in R. Hypothesis testing was done using *cor.test*, with multiple hypothesis testing correction using the Benjamini-Hochberg procedure using *p.adjust*. An FDR value of 0.05 is used as cutoff for significance pairwise correlations. Visualization was done using *pheatmap* package in R.

### Predictive modelling and evaluation

We choose candidate models based on previous research utilizing supervised learning with microbiome associated prediction tasks [28, 29, 31]. Specifically, we chose random forest (RF) [86], support vector machine with radial basis function kernel (SVM-RBF) [87], elastic net (EN) [88] and sparse partial least squares (SPLS) [89], which have all been shown to perform with high-dimensional predictors. These models also support linear and non-linear associations between the microbiome and the outcome of interest. Model fitting, parameter tuning, and evaluation were done using *caret* package in R [90]. Parallel processing was performed using the *doParallel* [91] and *parallel* packages.

We evaluate prediction performance by performing 100 repeats of 10-fold nested cross validation, whereby within each training fold is a separate 5-fold cross-validation procedure done to perform hyperparameter selection when appropriate with parameter grids modelled after Pasolli et al. [31]. For RF, we set the number of trees to be 500, and the number of features used in each decision tree to be the square root of the number of the original features. For SVM-RBF, we tuned across a grid for the regularization parameter *C* (values 2^−5^, 2^−3^, …, 2^15^) and the kernel width parameter γ (values 2^−15^, 2^−13^, …, 2^3^). For EN, we tuned over a grid of the regularization parameter λ and the L_1_ to L_2_ penalty ratio α, where for each α value (spaced by 0.1) between 0 (equivalent to a LASSO model) and 1 (equivalent to a ridge regression model), we evaluate 100 lambda values chosen by the *glmnet* procedure. For SPLS, we kept the concavity parameter κ constant at 0.5 while tuning the number of components *K* (values 1, 2, …, 10) and the thresholding parameter η (values 0.1,0.2, …, 0.9).

We utilize standard regression evaluation metrics include predictive R-squared (R^2^) and Spearman correlation coefficient (SCC).

These statistics were chosen due to their ability to capture two different aspects of the regression task. Predictive R^2^ captures the predicted residual sum of squares (PRESS) normalized by the total sum of squares of the outcome, thereby measuring predictive performance while also putting it into context of a naive, intercept only model. On the other hand, SCC quantifies the monotonic association between true and predicted values, providing perspective as to whether the predicted values can capture the overall trend of the outcome. Prior to evaluation, all metabolites were back transformed to their original scale. In order to perform comparisons between models across time points and metabolites as well as ascertaining the uncertainty of each evaluation metric, a Bayesian approach as presented in [92]. Specifically, a generalized Bayesian hierarchical linear model (with identity link and gaussian standard error) in the following form was fitted for each metabolite:

$$ EvaluationStatistic\sim Model+\left(1| repeat\right)+\left(1| repeat: fold\right) $$

This model assumes that the distribution of the evaluation statistic as a linear function of model assignment, with random intercepts varying among repeats and for folds within each repeat. Models were fitted using implementation in the R package *tidyposterior* [93] using default weakly informative priors as described in the *rstanarm* package [94]. Using this model, a predictive posterior mean and 95% credible interval can be generated. The posterior mean is then used to rank the best performing model for each metabolite according to the evaluation metric of interest. Ranks are then aggregated using the Borda method [95] to generate Borda scores. In detail, for each metabolite, 4 points are added to the top ranked model, 3 points to the second ranked model and so on. The model with the highest total points for each metric is the most performant model aggregated across all prediction tasks.

### Simulation design

Simulations were performed to examine the behavior of models under known association/null settings in order to validate findings. For the first simulation scenario, a linear association between genus-level taxonomic abundance and log transformed metabolite concentrations were simulated. The predictor matrix were bootstrapped resamples of the community matrix post data processing. *β* coefficient values were sampled from the standard normal distribution *N*(0, 1) values for each genus would have a probability *p* (0.05, 0.1, 0.5, 0.95) of being 0 which determines the sparsity of the coefficients (or the level of model saturation). We generate metabolite outcome values *Y* following the model

$$ Y={\beta}_0+ X\beta +\epsilon $$

where *X* is the *n* × *p* simulated taxonomic predictor matrix, *β* is the *p* × 1 previously defined coefficient vector, *ϵ* ∼ *N*(*μ* = 0, *σ* = *σ*_*ϵ*_) is the standard normal noise vector. Similar to Xiao et al. 2018 [96] and Shi et al. 2016 [97], we set all ${\beta}_0=\frac{6}{\surd 10}$ and ${\sigma}_{\epsilon }=\frac{\sigma \left({\beta}_0+ X\beta \right)}{SNR}$ where signal-to-noise ratio (SNR) are set at 0.5, 0.7, 3, 5 to simulate both situations where noise is higher than signal and vice versa. For each simulation setting, 100 data sets were generated.

For the second simulation scenario, null models were assessed through a permutation procedure using the *picante* package in R as described earlier. A total of 500 permutations was performed for each model.

To evaluate the predictive capacity of models for each simulation scenario, each data set was split into a train and test set (80% train; 20% test). Within each training set, a 10-fold cross validation procedure was employed to tune any hyperparameters. Similar evaluation metrics were assessed as described in the model fitting section.

### Metagenomic prediction with PICRUSt2

We conducted a PICRUSt2 (version 2.3.0_b) [98] analysis to investigate the potential relationship between the functional metagenome (obtained via *in sillico* predictions) and measurements of associated metabolites. We performed this analysis for metabolites obtained in the targeted data set. The PICRUSt2 pipeline was performed on pre-filtered ASV sequences and abundance tables using default settings. Snakemake was used to construct the computational pipeline [99].

After obtaining predicted MetaCyc pathway abundances, for each metabolite, we selected a subset of the pathways where the metabolite is a known product (accessed via MetaCyc SmartTables; 6-week samples: https://metacyc.org/group?id=biocyc13-50254-3822215614, 12-month samples: https://metacyc.org/group?id=biocyc13-50254-3822215614) and performed spearman correlation analysis with the measured metabolite abundances. For each significant correlation (significance level defined as q-values below 0.05 following the Benjamini-Hochberg procedure [100]), we profiled the relative contributions of the top five Genera. Relative contribution is calculated as total abundance of a pathway assigned to that Genus across all samples divided by the total abundance of the pathway across all samples.

Additionally, pairwise spearman correlation between all identified pathway abundances and targeted metabolite concentrations was also performed. Significance is defined similarly as FDR adjusted q-values below 0.05.

## Supplementary Information

**Additional file 1: **Contains supplementary notes (Notes 1–3), supplementary figures (Figures S1-S11), supplementary Table 1 and 2. **Figure S1.** Inter-omics Procrustes biplots comparing PCoA ordinations of untargeted metabolite profiles and taxonomic relative abundances for 6 weeks (left panels) (*n* = 158) and 12 months (right panels) (*n* = 262). Top panels present analyses based on ordinations from Euclidean distances of genus level abundances after centered log ratio transformation and Euclidean distances of arcsine square root transformed metabolite relative abundances. Bottom panel presents analyses based on generalized Unifrac distance of amplicon sequence variant (ASV) relative abundances and Euclidean distances of arcsine square root transformed metabolite relative abundances. **Figure S2.** Pairwise Spearman correlation of metabolite bins and genus-level taxonomic abundances for 6-weeks (panel A, *N* = 158) and 12-months (panel B, *N* = 282) infants. Left panel displays the overall correlation pattern, where non-significant correlations are not colored (false discovery rate (FDR) controlled q-value < 0.05). Right panel displays the same heatmap restricted to taxa and metabolites selected by the sparse CCA procedure. Additionally, correlation coefficient of the first sCCA variate pair, bootstrapped 95% confidence interval and permutation *p*-value are also reported. **Figure S3.** Comparative analysis predictive model performance across all metabolites in the untargeted dataset for both 6-weeks (n = 158) and 12-months (*n* = 282) timepoints. Top panel shows superimposed boxplots and violin plots of the distribution of predictive posterior mean for each evaluation metric across all 208 spectral bins. Bottom panels show aggregated model rankings for all metabolites using R-squared (left) and spearman correlation (right) using Borda scores (Methods). **Figure S4.** Results for positive (Panel A) and negative simulations (Panel B). Positive simulations were conducted based on bootstrapped resamples of the original data (12-month time point) and a normally distributed outcome vector which represented a log-transformed metabolite profile. Different levels of model saturation (horizontal, model sparsity (spar) at 0.05, 0.1, 0.5, 0.95) and effect sizes (vertical, signal-to-noise ratio (snr) at 0.5, 0.7, 3, 5) were assessed, with 100 data sets generated for each setting combination. Negative simulations were conducted based on permutations of the original data (12-month time point), with a total of 1000 permutations. Highly negative outliers were removed for the purposes of visualization. **Figure S5.** Inter-omics Procrustes biplots comparing PCoA ordinations of targeted metabolite profiles and taxonomic relative abundances in the sensitivity analyses for 6 weeks (left panels) (*n* = 65) and 12 months (right panels) (n = 65). Top panels present analyses based on ordinations from Euclidean distances of genus level abundances after centered log ratio transformation and Euclidean distances of arcsine square root transformed metabolite relative abundances. Bottom panel presents analyses based on generalized Unifrac distance of amplicon sequence variant (ASV) relative abundances and Euclidean distances of arcsine square root transformed metabolite relative abundances. **Figure S6.** Inter-omics Procrustes biplots comparing PCoA ordinations of untargeted metabolite bin relative concentrations and taxonomic relative abundances in the sensitivity analyses for 6 weeks (left panels) (*n* = 65) and 12 months (right panels) (*n* = 65). Top panels present analyses based on ordinations from Euclidean distances of genus level abundances after centered log ratio transformation and Euclidean distances of arcsine square root transformed metabolite relative abundances. Bottom panel presents analyses based on generalized Unifrac distance of amplicon sequence variant (ASV) relative abundances and Euclidean distances of arcsine square root transformed metabolite relative abundances. **Figure S7.** Pairwise spearman correlation of concentration-fitted targeted metabolite concentrations and genus-level taxonomic abundances for 6-weeks (panel A, *N* = 65) and 12-months (panel B, N = 65) infants in sensitivity analyses. Left panel displays the overall correlation pattern, where non-significant correlations are not colored (FDR controlled q-value < 0.05). Right panel displays the same heatmap restricted to taxa and metabolites selected by the sCCA procedure. Additionally, correlation coefficient of the first sCCA variate pair, bootstrapped 95% confidence interval (nboot = 5000) and permutation *p*-value (nperm = 1000) are also reported. **Figure S8.** Pairwise spearman correlation of untargeted metabolite bin relative concentrations and genus-level taxonomic abundances for 6-weeks (panel A, N = 65) and 12-months (panel B, N = 65) infants in sensitivity analyses. Left panel displays the overall correlation pattern, where non-significant correlations are not colored (FDR controlled q-value < 0.05). Right panel displays the same heatmap restricted to taxa and metabolites selected by the sCCA procedure. Additionally, correlation coefficient of the first sCCA variate pair, bootstrapped 95% confidence interval (nboot = 5000) and permutation p-value (nperm = 1000) are also reported. **Figure S9:** Spearman correlation coefficients and 95% confidence intervals of significant correlations (q-value < 0.05) between metabolite concentrations in the targeted data set and the abundances of pathways that produce them. Pathway abundances were obtained via PICRUSt2 predictions, with pathway-metabolite relationship retrieved from MetaCyc database. Both 6-week (*n* = 158) and 12-month (*n* = 282) samples are represented. **Figure S10.** Top five contributors at the Genus level for each significantly correlated pathway-metabolite pair obtained using observed metabolite concentrations and predicted pathway abundances (spearman correlation with q-value < 0.05). Panel A represents 6-week samples while panel B represents samples at 12-months. Relative contributions are calculated as the total number of copies of genes mapped to a pathway across all samples per Genus over the total number of gene copies assigned to that pathway. **Figure S11.** Heatmap representing overall spearman correlations between predicted pathway abundances (obtained via PICRUSt2) and metabolite concentrations in the targeted data set regardless of pathway-metabolite annotations. Both 6-week (n = 158) (Panel A) and 12-month (n = 282) (Panel B) samples are presented. Non-significant correlations (q-value > 0.05) are not colored. **Table S1.** Metabolites selected for targeted analysis and their potential biological functions. **Table S2.** Primers used for bacterial 16S rRNA gene sequencing.

**Additional file 2.** Pairs of microbes and targeted metabolites with significant spearman correlations at q-value < 0.05 with their correlation values for each time point.

**Additional file 3.** List of targeted metabolites with predictive R^2^ > 0 or spearman correlation > 0.3 for each model across all time points.

## Data Availability

The 16S rRNA gene sequencing datasets used in this study are stored in the National Center for Biotechnology Information (NCBI) Sequence Read Archive: http://www.ncbi.nlm.nih.gov/sra under accession number PRJNA296814. The raw and processed metabolomics data is available at the NIH Common Fund’s National Metabolomics Data Repository (NMDR) website, the Metabolomics Workbench, https://www.metabolomicsworkbench.org where it has been assigned Project ID PR001146. The data can be accessed directly via its project DOI: 10.21228/M8K69N. All intermediary analysis objects and scripts are available on GitHub.

## Abbreviations

NHBCS: New Hampshire Birth Cohort Study
NMR: Nuclear Magnetic Resonance
PCoA: Principal Coordinates Analysis
gUniFrac: Generalized Unique Fraction
ASV: Amplicon Sequence Variant
FDR: False Discovery Rate
sCCA/CCA: Sparse Canonical Correlation Analysis
SCFA: Short Chain Fatty Acids
RF: Random Forest
EN: Elastic Net
SVM-RBF: Support Vector Machines with Radial Basis kernel Function
SPLS: Sparse Partial Least Squares
CLR: Centered Log Ratio transformation
