# Supplementary material for: Associations between the gut microbiome and metabolome in early life
Source: BMC Microbiol. 2021 Aug 28;21:238. doi: 10.1186/s12866-021-02282-3 (PMC8400760; doi:10.1186/s12866-021-02282-3)
Supplement: Supplementary file 1 — Additional file 1: Contains supplementary notes (Notes 1–3), supplementary figures (Figures S1-S11), supplementary Table 1 and 2. Figure S1. Inter-omics Procrustes biplots comparing PCoA ordinations of untargeted metabolite profiles and taxonomic relative abundances for 6 weeks (left panels) (n = 158) and 12 months (right panels) (n = 262). Top panels present analyses based on ordinations from Euclidean distances of genus level abundances after centered log ratio transformation and Euclidean distances of arcsine square root transformed metabolite relative abundances. Bottom panel presents analyses based on generalized Unifrac distance of amplicon sequence variant (ASV) relative abundances and Euclidean distances of arcsine square root transformed metabolite relative abundances. Figure S2. Pairwise Spearman correlation of metabolite bins and genus-level taxonomic abundances for 6-weeks (panel A, N = 158) and 12-months (panel B, N = 282) infants. Left panel displays the overall correlation pattern, where non-significant correlations are not colored (false discovery rate (FDR) controlled q-value < 0.05). Right panel displays the same heatmap restricted to taxa and metabolites selected by the sparse CCA procedure. Additionally, correlation coefficient of the first sCCA variate pair, bootstrapped 95% confidence interval and permutation p-value are also reported. Figure S3. Comparative analysis predictive model performance across all metabolites in the untargeted dataset for both 6-weeks (n = 158) and 12-months (n = 282) timepoints. Top panel shows superimposed boxplots and violin plots of the distribution of predictive posterior mean for each evaluation metric across all 208 spectral bins. Bottom panels show aggregated model rankings for all metabolites using R-squared (left) and spearman correlation (right) using Borda scores (Methods). Figure S4. Results for positive (Panel A) and negative simulations (Panel B). Positive simulations were conducted based on bootstrapped [file 12866_2021_2282_MOESM1_ESM.docx]

**Supplementary Note 1: Microbe-metabolite participation in significant pairwise Spearman correlation**

Univariate pairwise Spearman correlations were performed to identify significant microbe-metabolite pairs. Significance was determined by the Spearman false discovery rate (FDR) threshold of 0.05 following a Benjamini-Hochberg multiple hypothesis testing procedure. At both time points a majority of genera and metabolites were significantly correlated, where at 6 weeks, 28 genera (65% of total genera) and 36 metabolites (100% of metabolites) were part of 516 significant correlations (16.6% of total pairwise comparisons) while at 12 months, 59 genera (81.9% of total genera) and 29 metabolites (80% of metabolites) were involved in 214 significant correlations (8.01% of total pairwise comparisons). This result also supported the observation that at 6 weeks the microbiome was marginally more associated with the metabolome compared to 12 months. Similar to sCCA results, untargeted data set showed a similar signal at both time points. Specifically, at 6 weeks, 37 genera (86% of genera) and 198 metabolite bins (95.1% of bins) were part of 1480 significant associations (16.5% of pairwise comparisons). Similarly, at 12 months, 67 genera (93% of genera) and 207 metabolite bins (99.5% of bins) were part of 1392 significant associations (9.2% of total pairwise comparisons).

**Supplementary Note 2: Sparse canonical correlation analysis selects microbes and metabolites important to the inter-omic correlation.**

Only a small subset of metabolites and microbes were selected (27% of taxa for both time points; 16.9% of metabolites at 6 weeks and 19.4% of metabolites at 12 months). At both time points, selected taxa belong to the Firmicutes, Actinobacteria and Proteobacteria phyla with Firmicutes being the most represented (58.3% of selected taxa at 6 weeks; 70% of selected taxa at 12 months). Actinobacteria was the second most selected phylum (25% of selected taxa) at 6 weeks while at 12 months it was Proteobacteria (30% of selected taxa). For metabolites, amino acids were the most represented metabolite class (Supplementary Table 1) (60% of selected metabolites at 6 weeks, 85% of selected metabolites at 12 months). 6-week samples demonstrated a larger diversity of metabolite classes, with additional representatives from carboxylic acids group, nucleotides and short chain fatty acids (SCFA) while at 12 months, the only non-amino-acid metabolite is uracil (of the nucleotide class). Across both time points, 3 genera (*Flavonifractor, Haemophilus* and *Acinetobacter* genera) and 5 metabolites (lysine, isoleucine, leucine, uracil, phenylalanine) were consistently selected. Surprisingly, in the untargeted analysis, nearly half of taxa and metabolites were selected at 6 weeks while the number remained more similar to the targeted analysis at 12 months (6 weeks: 46% of taxa and 42% of metabolite bins; 12 months: 13.8% of taxa and 17.3% of metabolite bins). However, the taxonomic distribution of those selected taxa remained similar, with Firmicutes being the most dominating phyla (60% of selected taxa at both time points). Additionally, for both time points, the sign of the sCCA loadings for selected variables were also concordant with patterns of negative and positive correlation via univariate Spearman correlations (Figure 3, right panels). Notably, all selected metabolites contain negative loadings for both time points, with the majority of selected pairwise correlation to be negative (6 weeks: 76.6% of selected pairwise comparisons, 12 months: 60.7% of selected pairwise comparisons). This pattern is replicated in the untargeted data set as well (6 weeks: 61.3% of selected pairwise comparisons, 12 months: 70% of selected pairwise comparisons).

**Supplementary Note 3: Prediction results.**

Under R^2^, at 6 weeks only 8 (22.2%) metabolites (Butyrate, Glycerol, Isobutyrate, Isoleucine, Leucine, Methionine, Phenylalanine and Tyrosine) were predictable, with a mean of 4.85% and a maximum of 11.8% (Butyrate using EN). At 12 months, only 14 (38.9%) of metabolites (Butyrate, Formate, Inosine, Isobutyerate, Isoleucine, Lactate, Leucine, Methionine, Phenylalanine, Propionate, Propylene glycol, Tyrosine, Uracil, Valine) were predictable, with a mean of 4.81% and a maximum of 8.7% (Propylene Glycol using RF). When looking at the average R^2^ across all metabolites, performance was not good (-5.6% at 6 weeks; -3.07% at 12 months). This negative R^2^ value implies that the predicted model performs worse than the naïve, intercept only model. Conversely, correlative performance was much better. At 6 weeks 26 metabolites (83%) were predictable, with a mean correlation of 0.344 and a maximum of 0.669 (Butyrate using EN). Similarly, at 12 months all 36 targeted metabolites were predictable, with a mean of 0.265 and a maximum of 0.549 (Succinate using EN). Using the SCC cutoff of 0.3 as criteria for well predicted metabolites, many metabolites at 6 weeks were still retained (25 metabolites - 69.4%). Conversely, at 12 months, only 13 metabolites remained to be well predicted (38.9%). On average, performance based on SCC was good with a mean SCC value of 0.339 at 6 weeks and 0.249 at 12 months.

In the untargeted analysis looking at the entire metabolome, performance was much better for both metrics. Under R^2^, 116 (56.7%) of metabolite bins were predictable at a maximum of 42.7% (Bin 33 using SPLS) and a mean of 16.7% at 6 weeks while at 12 months, 94 (45.1%) of metabolite bins were ell predicted at a maximum of 22.7% (Bin 16 using SVM) and a mean of 8.19%. The overall average across all metabolites is 3.91% (6 weeks) and -0.59% (12 months). This trend was similarly observed using SCC, as all all 208 metabolites bins were predictable for both time points (using SCC = 0 as the threshold). Specifically, of the predictable metabolites at 6 weeks the maximum value was 0.687 (Bin 32 using EN) with a mean of 0.352 while at 12 months, the maximum value was 0.53 (Bin 16 using SVM) and a mean of 0.253. Using the SCC cutoff of 0.3 as above, at 6 weeks 120 metabolite bins (57% of bins) were well predicted while at 12 months only 60 (28.8% of bins) were well predicted.


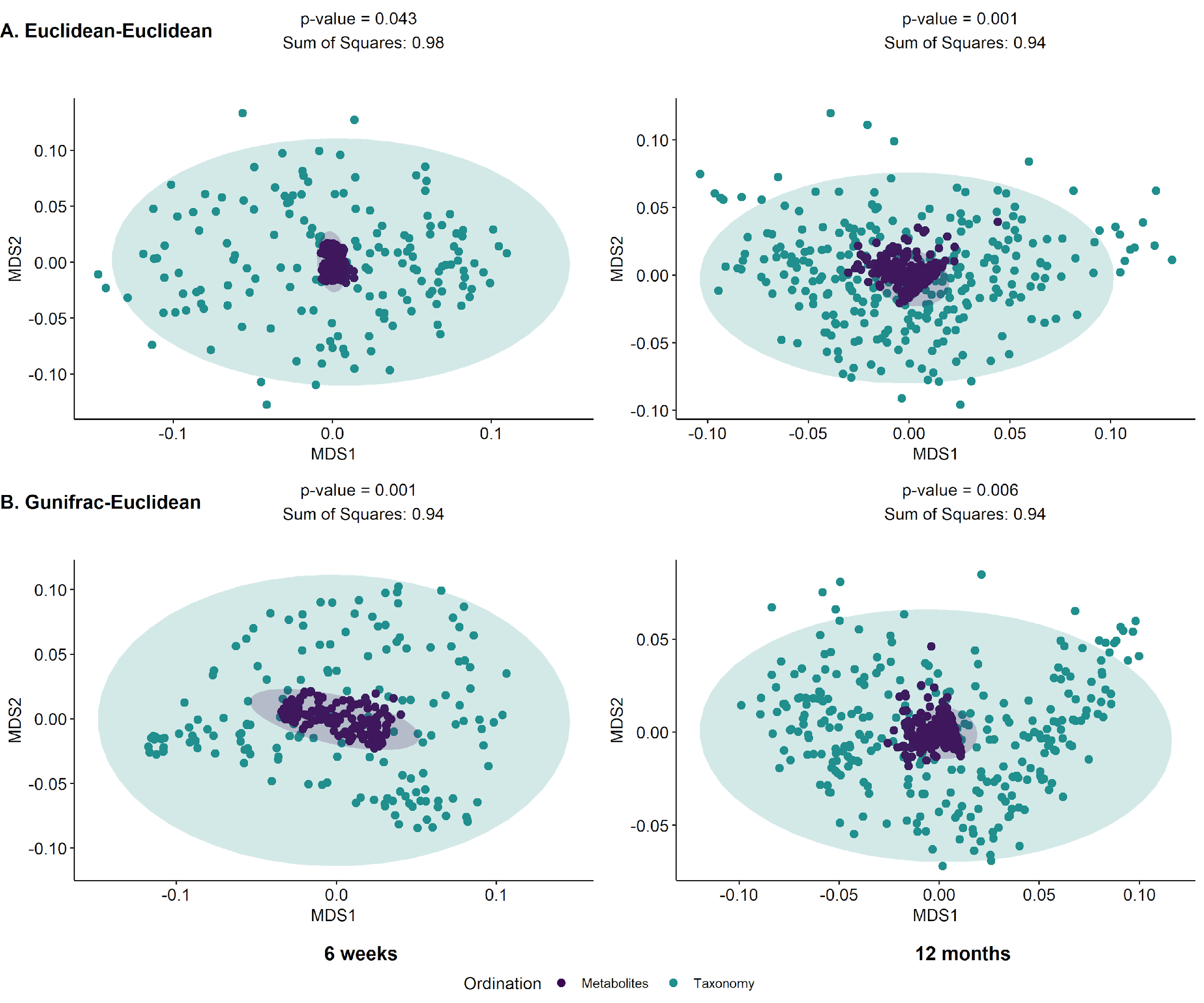


**Figure S1.** Inter-omics Procrustes biplots comparing PCoA ordinations of untargeted metabolite profiles and taxonomic relative abundances for 6 weeks (left panels) (n = 158) and 12 months (right panels) (n = 262). Top panels present analyses based on ordinations from Euclidean distances of genus level abundances after centered log ratio transformation and Euclidean distances of arcsine square root transformed metabolite relative abundances. Bottom panel presents analyses based on generalized Unifrac distance of amplicon sequence variant (ASV) relative abundances and Euclidean distances of arcsine square root transformed metabolite relative abundances.


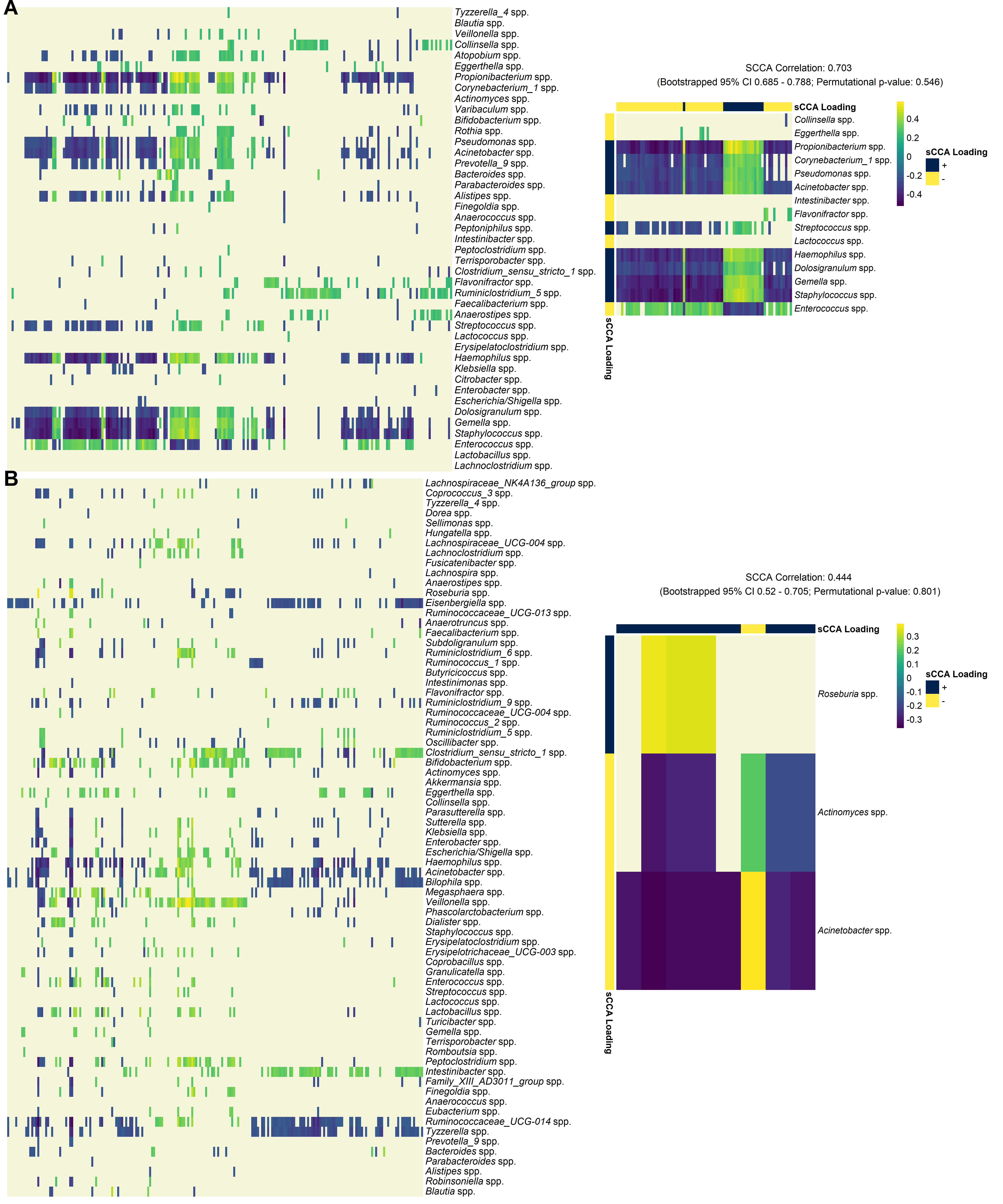


**Figure S2.** Pairwise Spearman correlation of metabolite bins and genus-level taxonomic abundances for 6-weeks (panel A, N = 168) and 12-months (panel B, N = 282) infants. Left panel displays the overall correlation pattern, where non-significant correlations are not colored (false discovery rate (FDR) controlled q-value < 0.05). Right panel displays the same heatmap restricted to taxa and metabolites selected by the sparse CCA procedure. Additionally, correlation coefficient of the first sCCA variate pair, bootstrapped 95% confidence interval and permutation p-value are also reported.


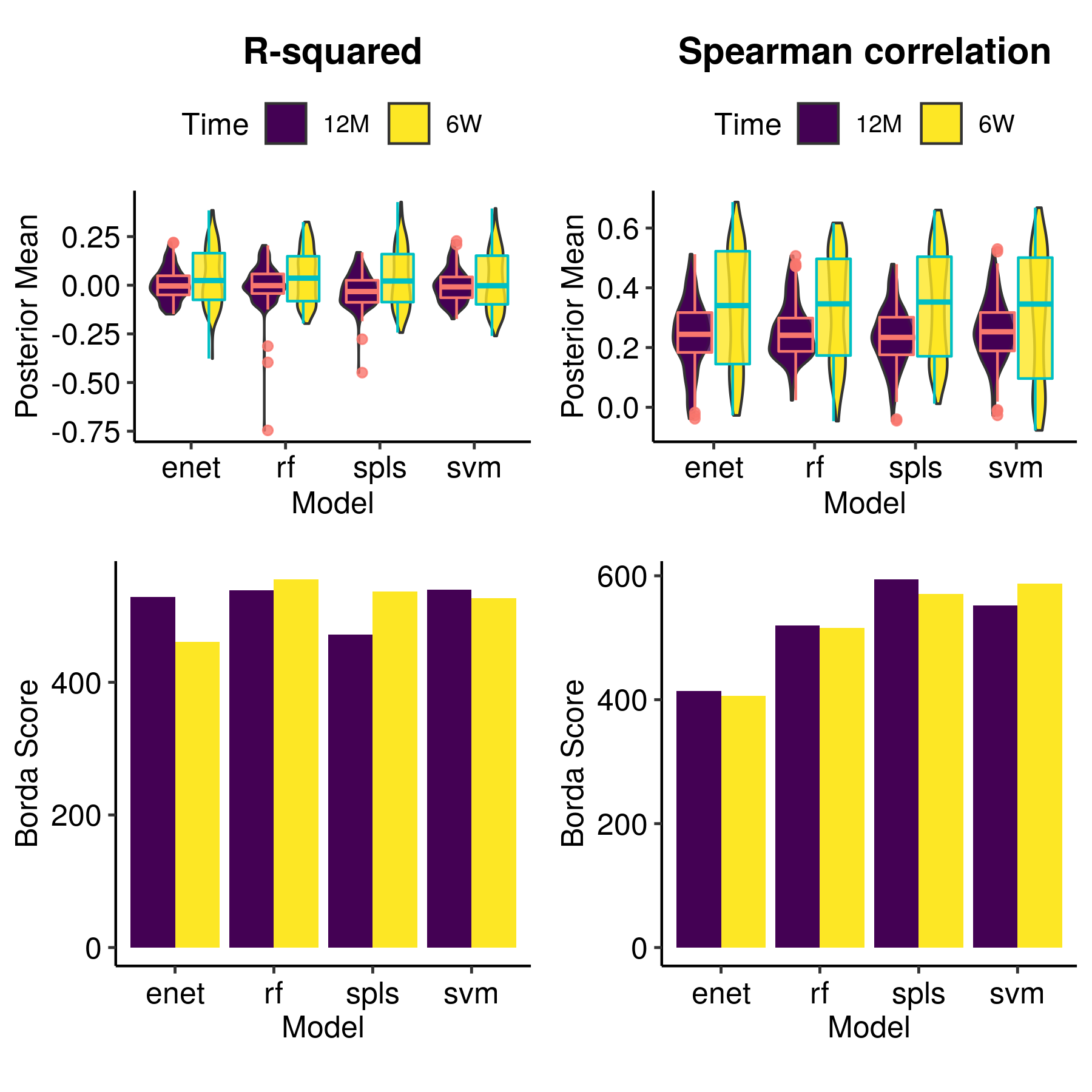


**Figure S3.** Comparative analysis predictive model performance across all metabolites in the untargeted dataset for both 6-weeks (n = 158) and 12-months (n = 282) timepoints. Top panel shows superimposed boxplots and violin plots of the distribution of predictive posterior mean for each evaluation metric across all 208 spectral bins. Bottom panels show aggregated model rankings for all metabolites using R-squared (left) and spearman correlation (right) using Borda scores (Methods).

**
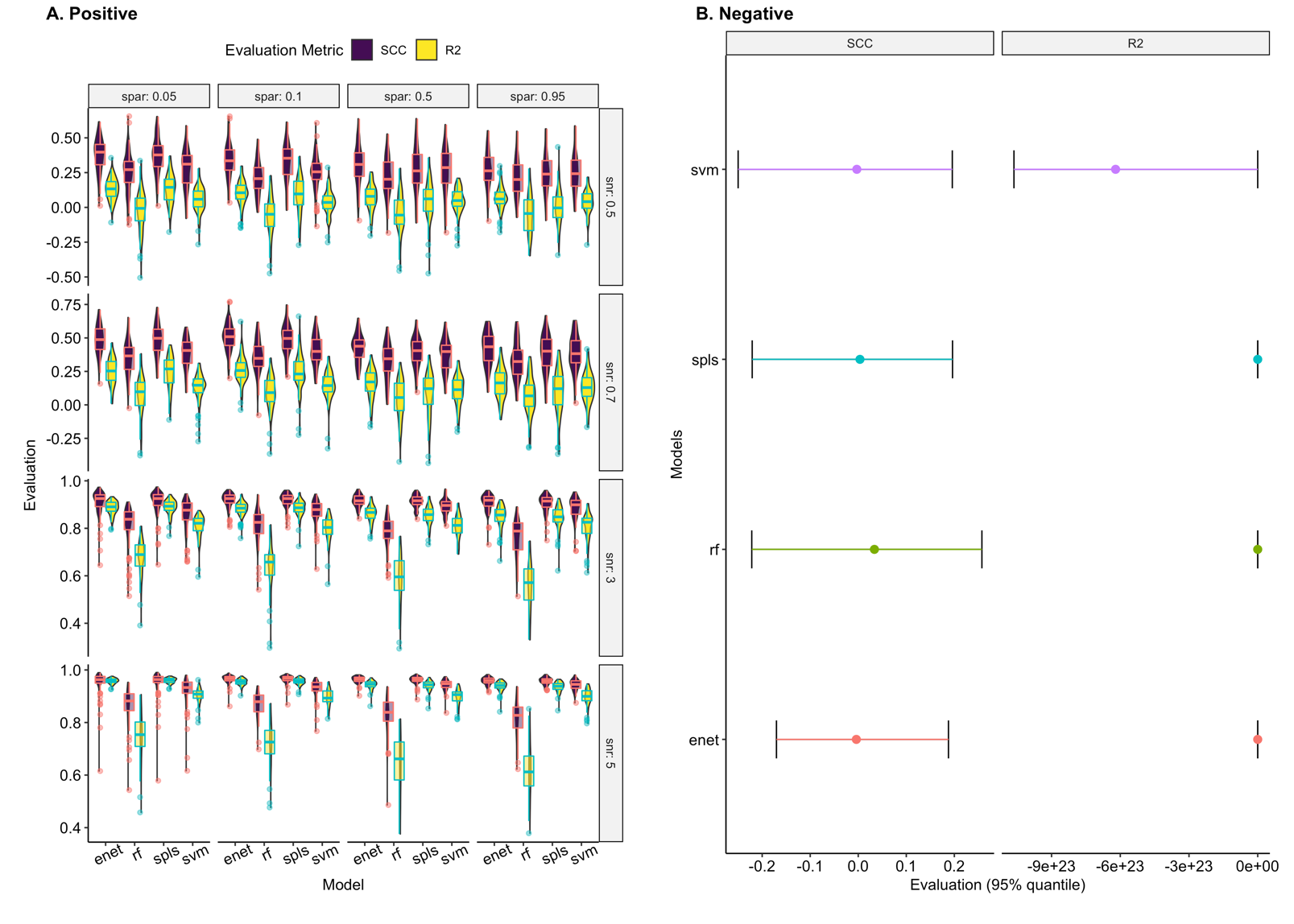
**

**Figure S4.** Results for positive (Panel A) and negative simulations (Panel B). Positive simulations were conducted based on bootstrapped resamples of the original data (12-month time point) and a normally distributed outcome vector which represented a log-transformed metabolite profile. Different levels of model saturation (horizontal, model sparsity (spar) at 0.05, 0.1, 0.5, 0.95) and effect sizes (vertical, signal-to-noise ratio (snr) at 0.5, 0.7, 3, 5) were assessed, with 100 data sets generated for each setting combination. Negative simulations were conducted based on permutations of the original data (12-month time point), with a total of 1000 permutations. Highly negative outliers were removed for the purposes of visualization.


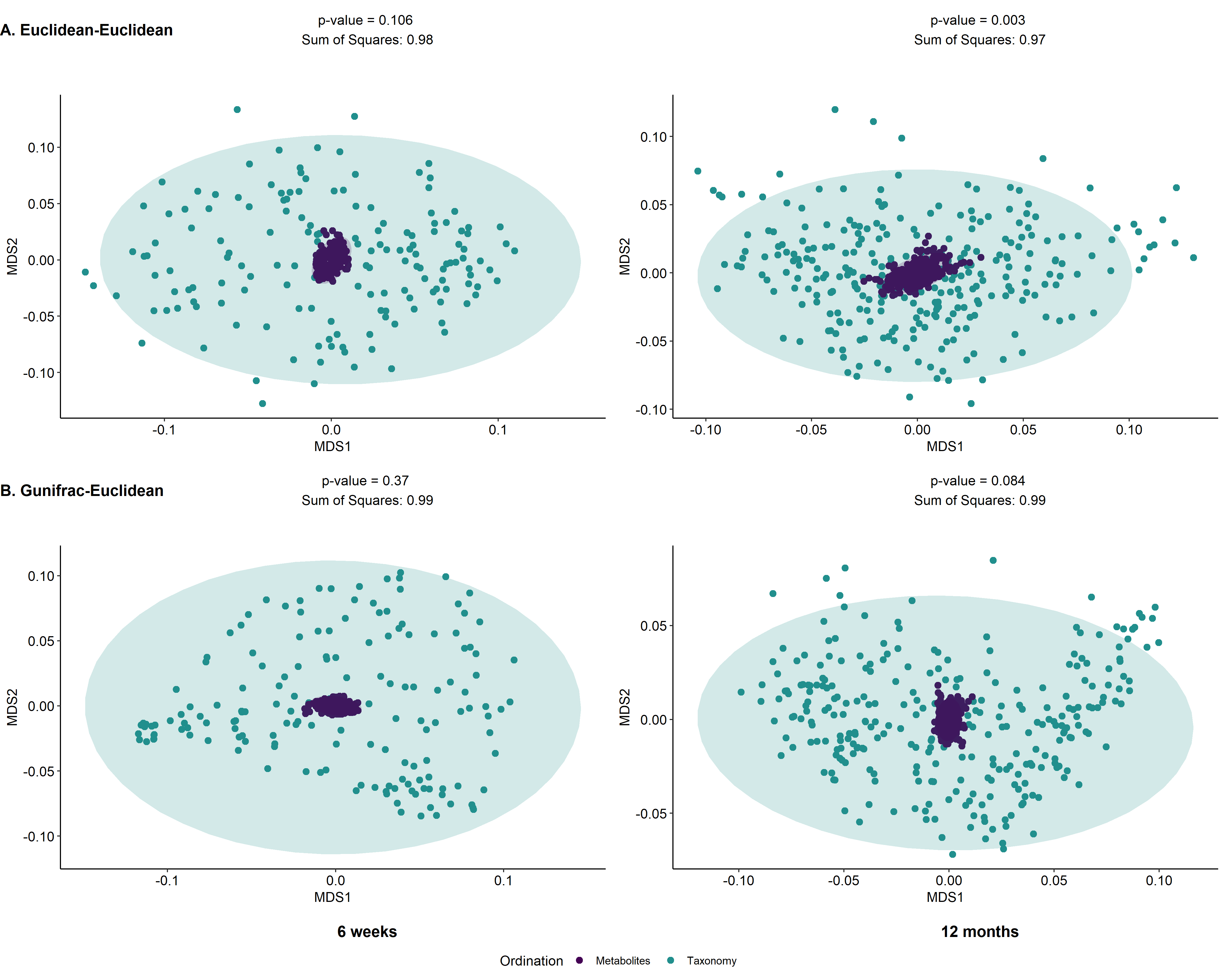


**Figure S5.** Inter-omics Procrustes biplots comparing PCoA ordinations of targeted metabolite profiles and taxonomic relative abundances in the sensitivity analyses for 6 weeks (left panels) (n = 65) and 12 months (right panels) (n = 65). Top panels present analyses based on ordinations from Euclidean distances of genus level abundances after centered log ratio transformation and Euclidean distances of arcsine square root transformed metabolite relative abundances. Bottom panel presents analyses based on generalized Unifrac distance of amplicon sequence variant (ASV) relative abundances and Euclidean distances of arcsine square root transformed metabolite relative abundances.


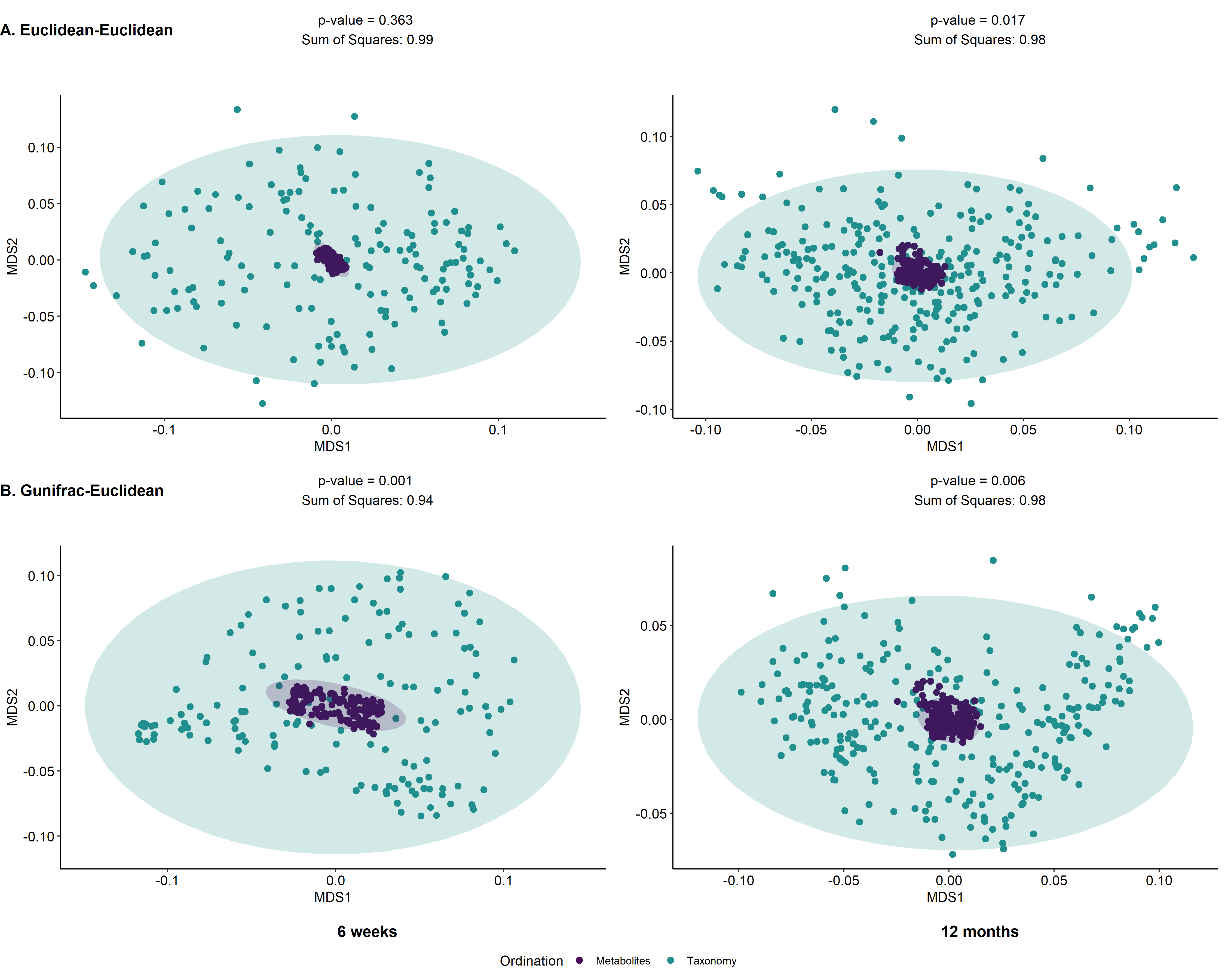


**Figure S6.** Inter-omics Procrustes biplots comparing PCoA ordinations of untargeted metabolite bin relative concentrations and taxonomic relative abundances in the sensitivity analyses for 6 weeks (left panels) (n = 65) and 12 months (right panels) (n = 65). Top panels present analyses based on ordinations from Euclidean distances of genus level abundances after centered log ratio transformation and Euclidean distances of arcsine square root transformed metabolite relative abundances. Bottom panel presents analyses based on generalized Unifrac distance of amplicon sequence variant (ASV) relative abundances and Euclidean distances of arcsine square root transformed metabolite relative abundances.


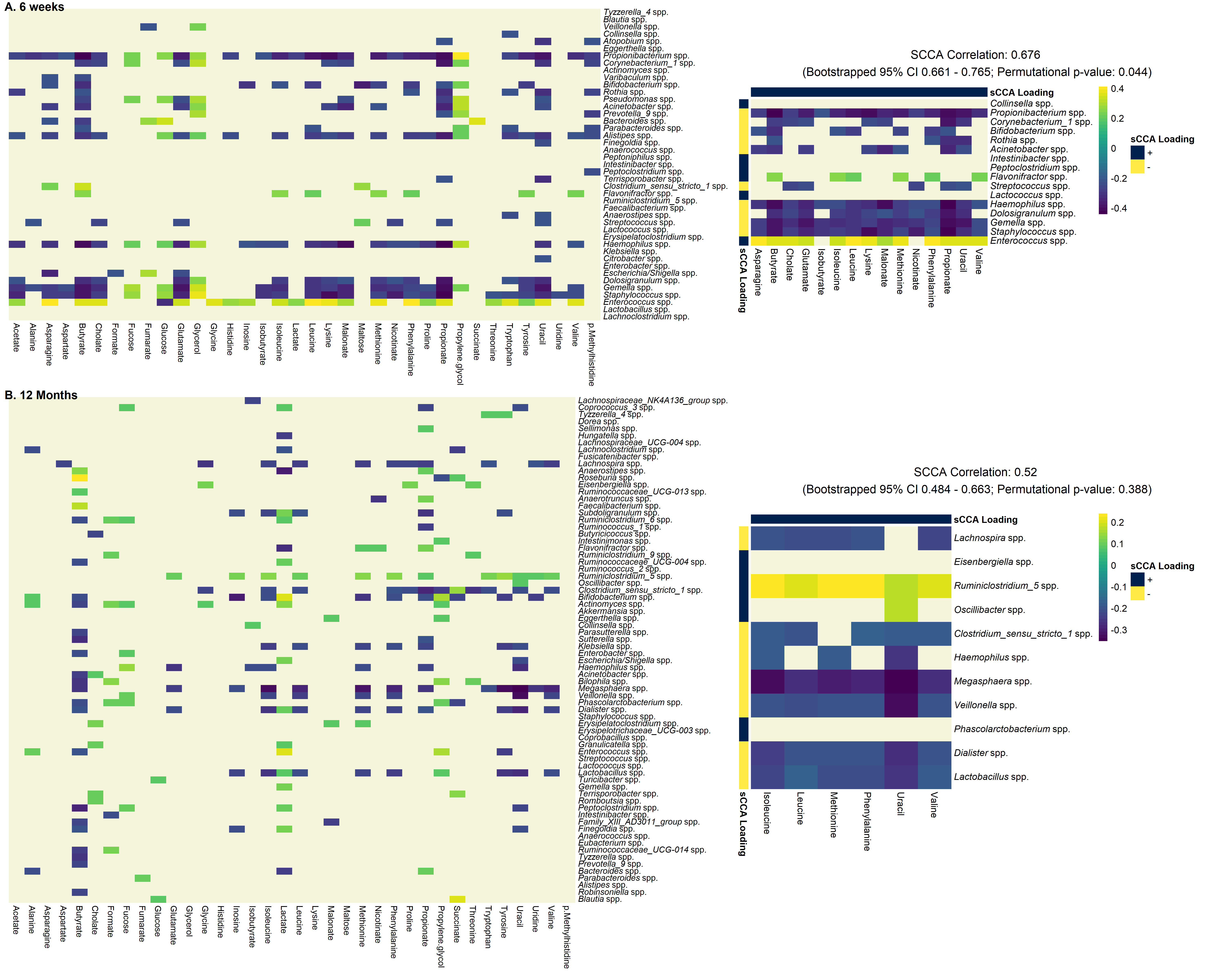


**Figure S7.** Pairwise spearman correlation of concentration-fitted targeted metabolite concentrations and genus-level taxonomic abundances for 6-weeks (panel A, N = 65) and 12-months (panel B, N = 65) infants in sensitivity analyses. Left panel displays the overall correlation pattern, where non-significant correlations are not colored (FDR controlled q-value < 0.05). Right panel displays the same heatmap restricted to taxa and metabolites selected by the sCCA procedure. Additionally, correlation coefficient of the first sCCA variate pair, bootstrapped 95% confidence interval (nboot = 5000) and permutation p-value (nperm = 1000) are also reported.


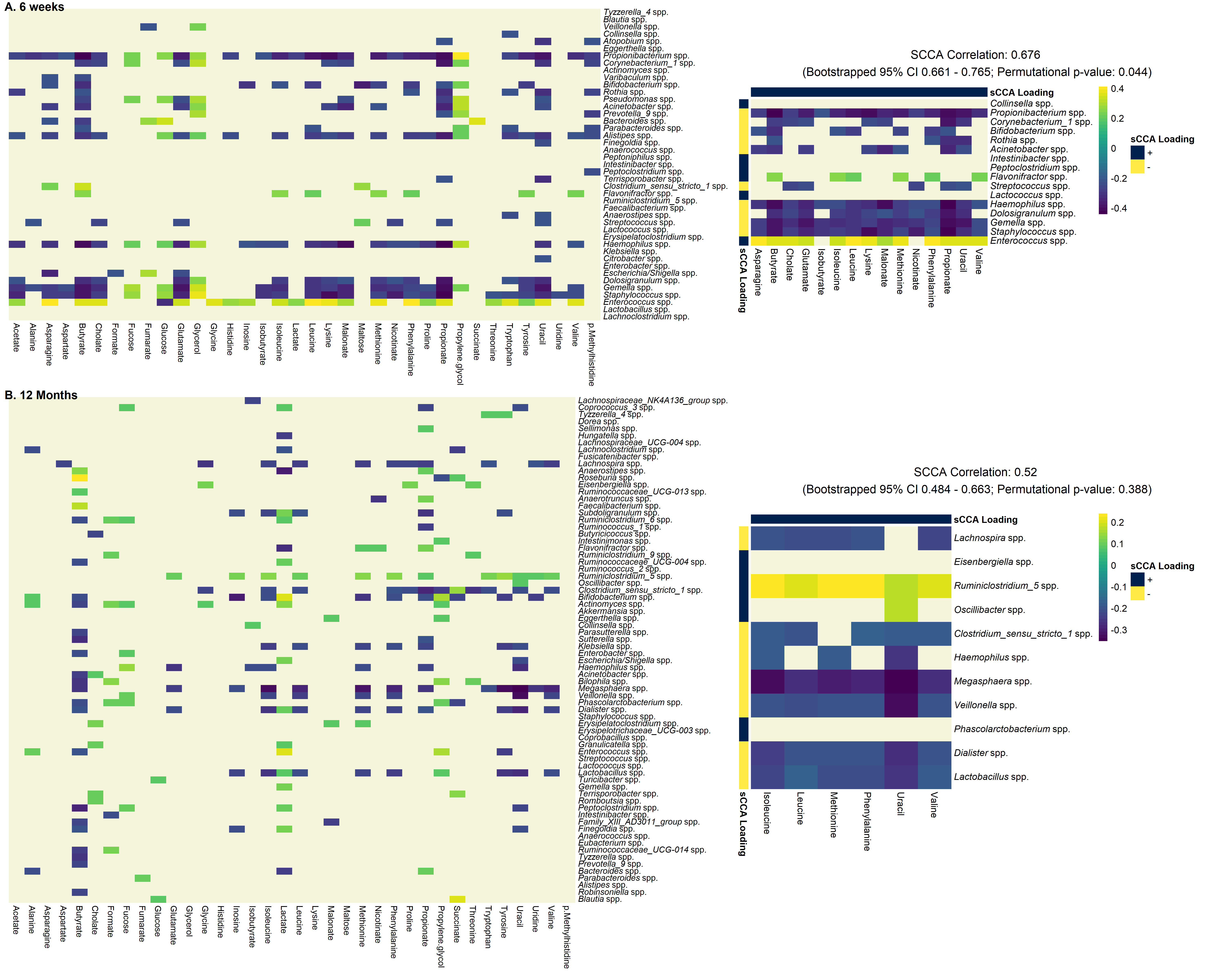


**Figure S8.** Pairwise spearman correlation of untargeted metabolite bin relative concentrations and genus-level taxonomic abundances for 6-weeks (panel A, N = 65) and 12-months (panel B, N = 65) infants in sensitivity analyses. Left panel displays the overall correlation pattern, where non-significant correlations are not colored (FDR controlled q-value < 0.05). Right panel displays the same heatmap restricted to taxa and metabolites selected by the sCCA procedure. Additionally, correlation coefficient of the first sCCA variate pair, bootstrapped 95% confidence interval (nboot = 5000) and permutation p-value (nperm = 1000) are also reported.


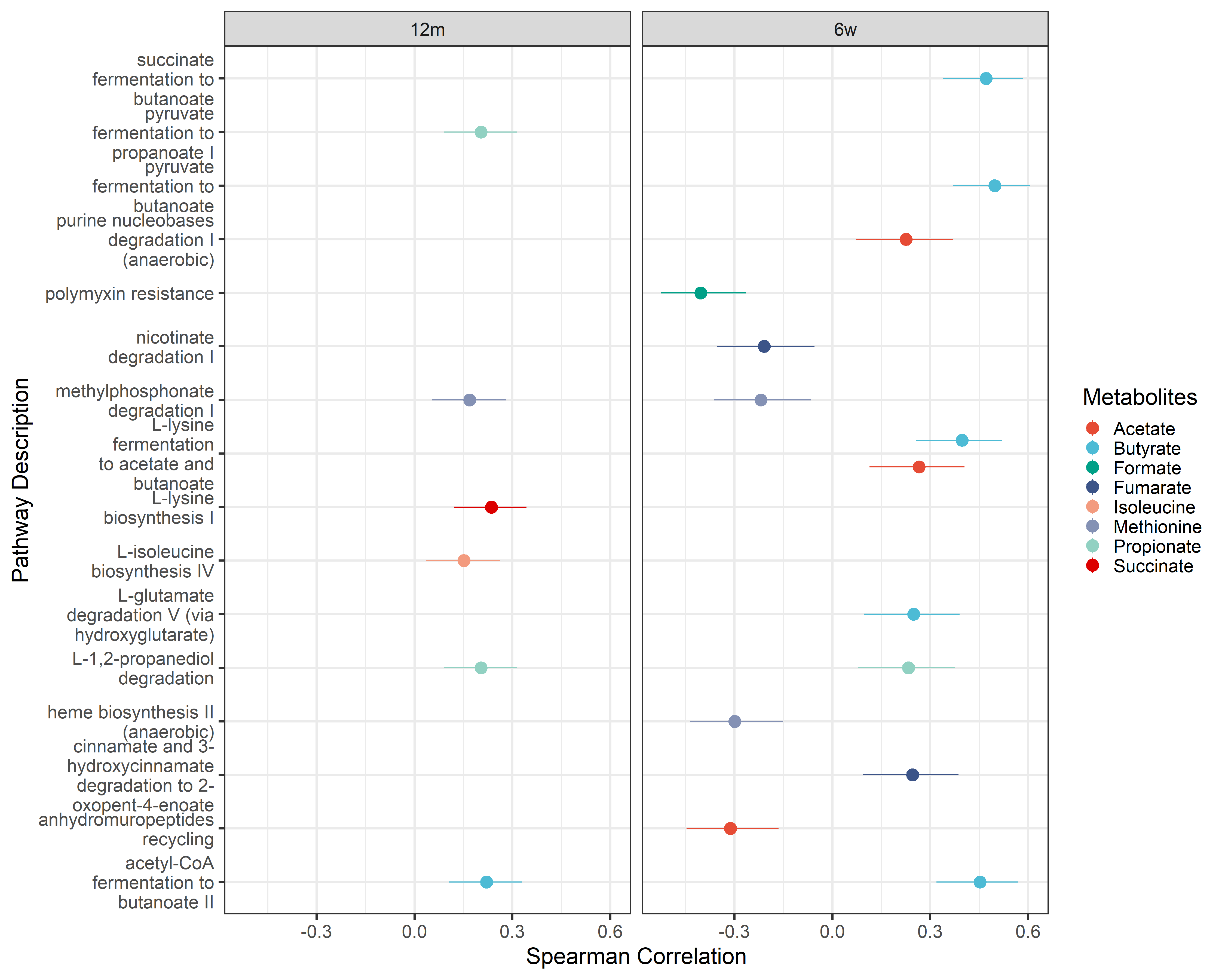


**Figure S9:** Spearman correlation coefficients and 95% confidence intervals of significant correlations (q-value < 0.05) between metabolite concentrations in the targeted data set and the abundances of pathways that produce them. Pathway abundances were obtained via PICRUSt2 predictions, with pathway-metabolite relationship retrieved from MetaCyc database. Both 6-week (n = 158) and 12-month (n = 282) samples are represented.


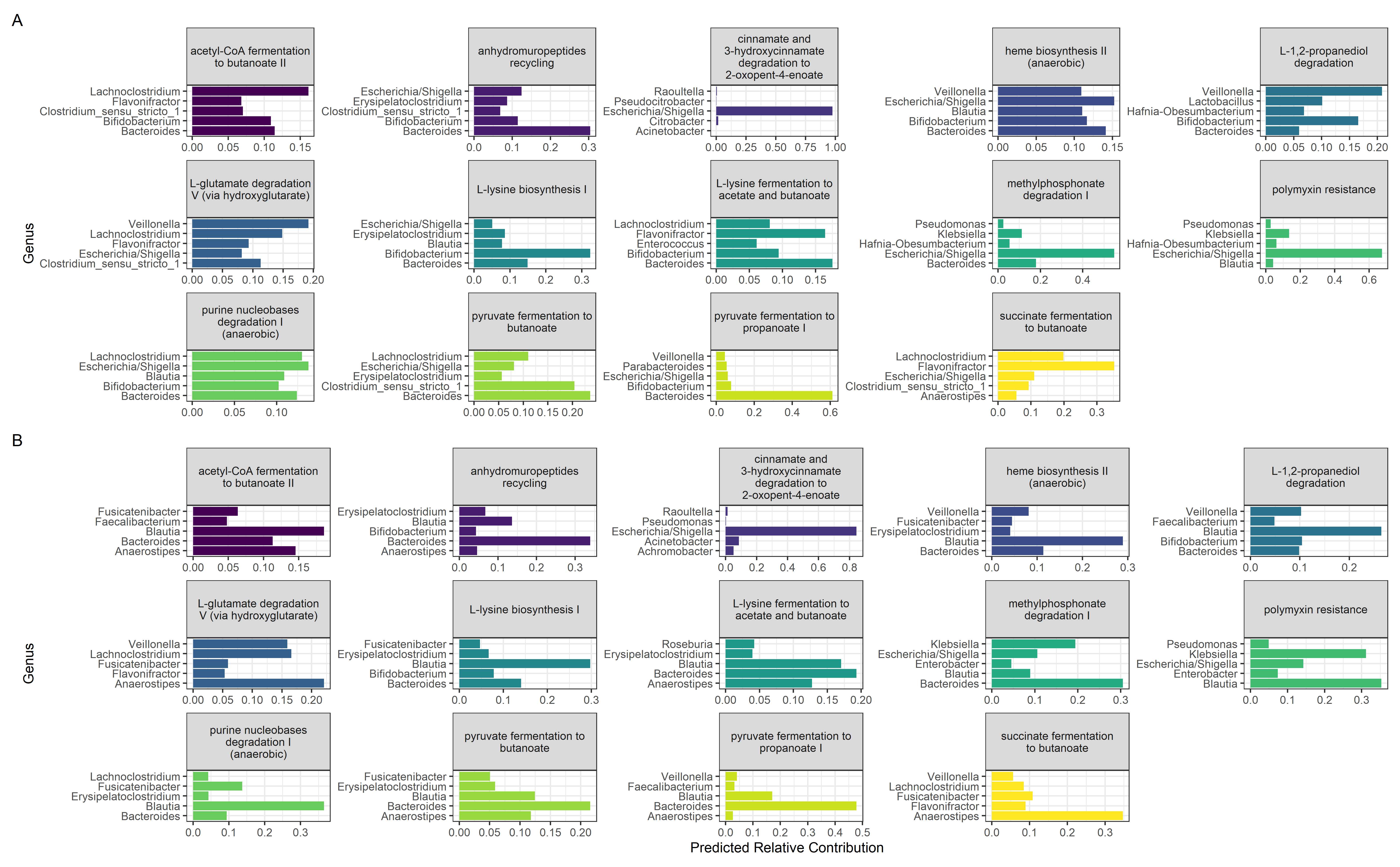
**Figure S10.** Top five contributors at the Genus level for each significantly correlated pathway-metabolite pair obtained using observed metabolite concentrations and predicted pathway abundances (spearman correlation with q-value < 0.05). Panel A represents 6-week samples while panel B represents samples at 12-months. Relative contributions are calculated as the total number of copies of genes mapped to a pathway across all samples per Genus over the total number of gene copies assigned to that pathway.

**
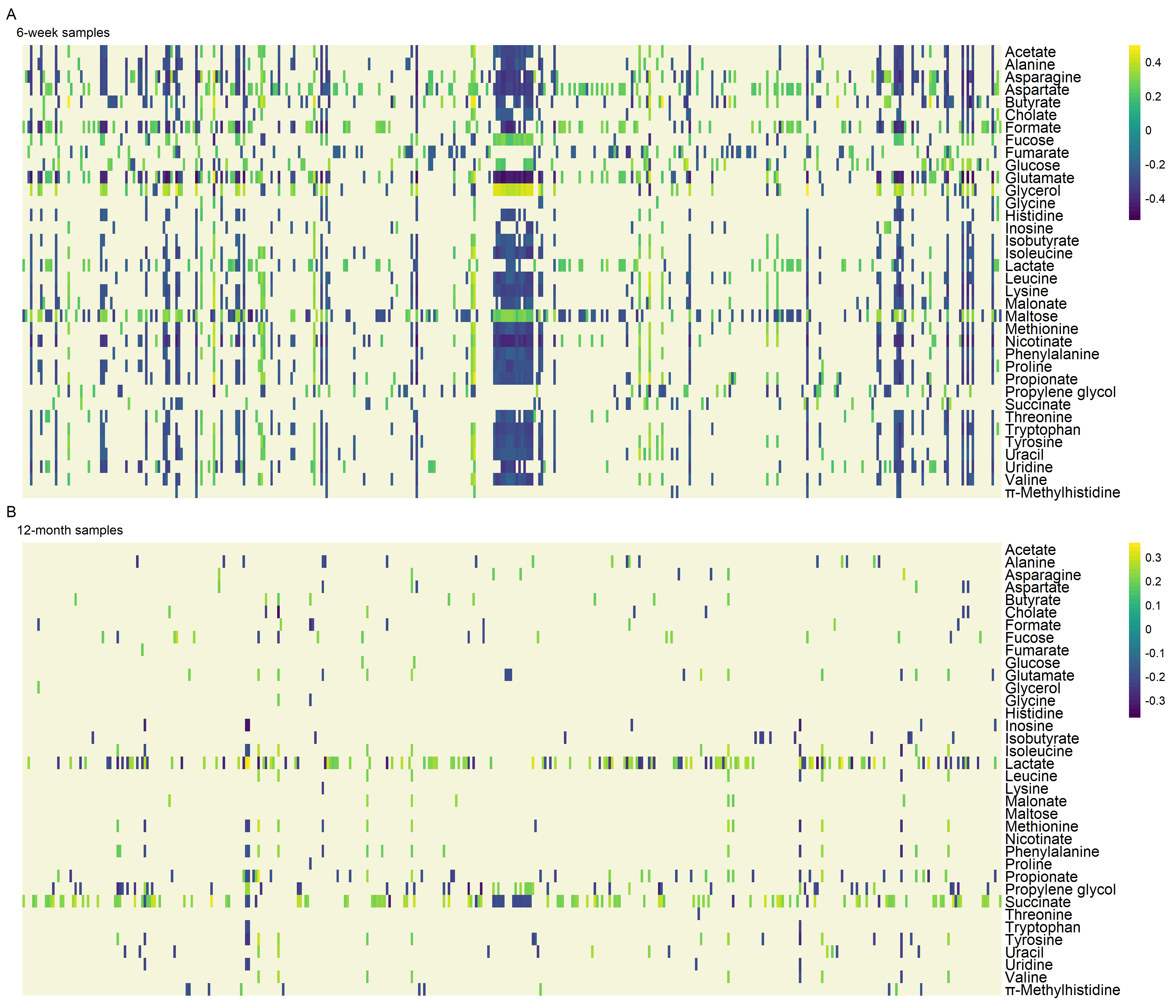
**

**Figure S11.** Heatmap representing overall spearman correlations between predicted pathway abundances (obtained via PICRUSt2) and metabolite concentrations in the targeted data set regardless of pathway-metabolite annotations. Both 6-week (n = 158) (Panel A) and 12-month (n = 282) (Panel B) samples are presented. Non-significant correlations (q-value > 0.05) are not colored.

**Table S1. Metabolites selected for targeted analysis and their potential biological functions.**

| **Metabolite class** | **Metabolites** | **Examples of associated**  **microbes** | **Potential**  **biological functions** | **HMDB ID*** |
| --- | --- | --- | --- | --- |
| Short chain fatty acids (SCFA) | Acetate  Butyrate  Isobutyrate Propionate | *Faecalibacterium, Eubacterium, Roseburia, Clostridia* clusters IV and XIVa [1] | Fermented in the colon from dietary complex carbohydrates, assist in regulating host immune functionality, act as substrate for cellular activities, limit growth of pathogenic species, promote integrity of the mucosal lining. | HMDB00042  HMDB00039  HMDB01873  HMDB00237 |
| Amino acids & derivatives | Alanine  Asparagine  Aspartate  Glutamate  Glycine  Histidine  Isoleucine  Leucine  Lysine  Methionine  Phenylalanine  Proline  Threonine  Tryptophan  Tyrosine  Valine  π-Methylhistidine* | Proteobacteria phylum; Bacili class; *Clostridium* and *Bifidobacterium* genera [2], lactobacilli, enterococci, and streptococci families [3]; *Faecalibacterium prausnitzii* species [4] | Catabolized to form other end products such as SCFAs, branched chain fatty acids (BCFAs) and other compounds [5]. For example, the catabolism of methionine results in methanethiol and hydrogen sulfide [2]; catabolism of histidine can produce histamine, which can inhibit the production of pro-inflammatory cytokines as well as act as a neurotransmitter [6]; catabolism of lysine can produce cadaverine [3], which is associated with ulcerative colitis [7]; catabolism of tryptophan and tyrosine can produce tryptamine, which is a neurotransmitter involved in intestinal motility and immune function [8]. | HMDB00161  HMDB00168  HMDB00191  HMDB00148  HMDB00123  HMDB00177  HMDB00172  HMDB00687  HMDB00182  HMDB00696  HMDB00159  HMDB00162  HMDB00167  HMDB00929  HMDB00158  HMDB00883  HMDB00479 |
| Bile acids | Cholate | Inhibits Bacterioidetes and Actinobacteria phyla, expansion of Firmicutes phylum, *Blautia, Clostridium* and *Ruminococcus spp.* [9, 10] | Bile acid are involved in absorption of fats and lipid-soluble vitamins [1], bile acid byproducts of microbial origins can bind and activate host nuclear receptors and act as endocrine signaling molecules [11, 12], which is found to be associated with cancer [13]. | HMDB00619 |
| Carbohydrates | Fucose  Glucose  Glycerol  Maltose | *Bacteroides thetaiotaomicron* (possesses 260 glycoside hydrolases in its genome [14])*, B. Fragilis, Ruminococcaceae spp.* | Humans rely mostly on gut commensals for breaking down complex carbohydrates [5]. Bacteria also take in complex carbohydrates for additional purposes. For example, fucose participate in the fucosylation of bacterial glycans, increasing fitness for both pathogenic and commensal microbes through host mimicry; facilitate promotion of useful bacterial species and metabolites, suppress virulence genes [15]. Similarly, gut microbe can metabolize glycerol into reuterin, which is an antimicrobial multicomponent system [16]. | HMDB00174  HMDB00122  HMDB00131  HMDB00163 |
| Carboxylic and dicarboxylic acids | Formate  Fumarate  Malonate  Succinate  Lactate | Bloom of Enterobacteriaceae phylum. Others include Verrucomicrobia phylum. Producers from pyruvate catabolism includes *Bacteroides* and *Clostridia* genera [17]. Specifically for lactate, *Lactobacilli, Lactococci, Streptococci, Leuconostoc* and *Pediococci* genera [18] | Often used as terminal electron acceptors for bacterial anaerobic respiration [19] and is produced by the microbiota itself [20] and a highly competitive resource especially if induced by antibiotics [21]. The presence and activation of enzymes associated with oxidizing these acids are markers of inflammation such as in the case of formate [17]. Notably, lactate is an important component in designing lactic acid bacteria probiotics which can modulate intestinal immunity and provide a protective effect against infection [22]. | HMDB00142  HMDB00134  HMDB00691  HMDB00254  HMDB00190 |
| Nucleosides | Inosine  Uridine  Uracil | *Anaerococcus, Peptoniphilus, Fusobacterium, Lactobacillus* genera [23] | Can play an important role in immune response at the neonatal stage [24], supplementing the process of enterocyte proliferation, maturation and apoptosis of intestinal cells [25]. | HMDB00195  HMDB00296  HMDB00300 |
| Vitamins | Nicotinate (Vitamin B3) | Lactic-acid commensal bacteria such as *Bifidobacterium bifidum*, B. *longum, B. breve*, and *B. adolescentis*. [26] | Microbiome has been shown to both metabolize dietary B vitamins as well as produce them through folate metabolism [26]. It is well known that vitamin Bs are essential micronutrients that are precursors to important enzymes in humans. | HMDB01488 |
| Alcohols | Propylene Glycol | Firmicutes and Lachnospiraceae phyla, *Dorea, Robinsonella* and *Roseburia* genera [27]. | A solvent involved in propanoate metabolism resulting in propanal (through KEGG [28]), which have been shown to be associated with inflammatory bowel disease. | HMDB01881 |

* π-Methylhistidine is a histidine derivative

* HMDB ID: Human metabolome database ID

**Table S2**: Primers used for bacterial 16S rRNA gene sequencing

| Bacterial v4v5 Illumina Miseq Primer |  |
| --- | --- |
| Forward Primer (518F) | CCAGCAGCYGCGGTAAN |
| Reverse Primers (926R) | CCGTCAATTCNTTTRAGT CCGTCAATTTCTTTGAGT CCGTCTATTCCTTTGANT |

**References**

1. Nicholson JK, Holmes E, Kinross J, Burcelin R, Gibson G, Jia W, et al. Host-Gut Microbiota Metabolic Interactions. Science. 2012;336:1262–7.

2. Portune KJ, Beaumont M, Davila A-M, Tomé D, Blachier F, Sanz Y. Gut microbiota role in dietary protein metabolism and health-related outcomes: The two sides of the coin. Trends Food Sci Technol. 2016;57:213–32.

3. Pugin B, Barcik W, Westermann P, Heider A, Wawrzyniak M, Hellings P, et al. A wide diversity of bacteria from the human gut produces and degrades biogenic amines. Microb Ecol Health Dis. 2017;28:1353881.

4. Li M, Wang B, Zhang M, Rantalainen M, Wang S, Zhou H, et al. Symbiotic gut microbes modulate human metabolic phenotypes. Proc Natl Acad Sci. 2008;105:2117–22.

5. Oliphant K, Allen-Vercoe E. Macronutrient metabolism by the human gut microbiome: major fermentation by-products and their impact on host health. Microbiome. 2019;7:91.

6. Thomas CM, Hong T, van Pijkeren JP, Hemarajata P, Trinh DV, Hu W, et al. Histamine derived from probiotic Lactobacillus reuteri suppresses TNF via modulation of PKA and ERK signaling. PloS One. 2012;7:e31951.

7. Le Gall G, Noor SO, Ridgway K, Scovell L, Jamieson C, Johnson IT, et al. Metabolomics of fecal extracts detects altered metabolic activity of gut microbiota in ulcerative colitis and irritable bowel syndrome. J Proteome Res. 2011;10:4208–18.

8. Gao J, Xu K, Liu H, Liu G, Bai M, Peng C, et al. Impact of the Gut Microbiota on Intestinal Immunity Mediated by Tryptophan Metabolism. Front Cell Infect Microbiol. 2018;8:13.

9. Ridlon JM, Kang DJ, Hylemon PB, Bajaj JS. Bile Acids and the Gut Microbiome. Curr Opin Gastroenterol. 2014;30:332–8.

10. Islam KBMS, Fukiya S, Hagio M, Fujii N, Ishizuka S, Ooka T, et al. Bile acid is a host factor that regulates the composition of the cecal microbiota in rats. Gastroenterology. 2011;141:1773–81.

11. Kawamata Y, Fujii R, Hosoya M, Harada M, Yoshida H, Miwa M, et al. A G protein-coupled receptor responsive to bile acids. J Biol Chem. 2003;278:9435–40.

12. Hylemon PB, Zhou H, Pandak WM, Ren S, Gil G, Dent P. Bile acids as regulatory molecules. J Lipid Res. 2009;50:1509–20.

13. Yoshimoto S, Loo TM, Atarashi K, Kanda H, Sato S, Oyadomari S, et al. Obesity-induced gut microbial metabolite promotes liver cancer through senescence secretome. Nature. 2013;499:97–101.

14. Xu J, Bjursell MK, Himrod J, Deng S, Carmichael LK, Chiang HC, et al. A genomic view of the human-Bacteroides thetaiotaomicron symbiosis. Science. 2003;299:2074–6.

15. Pickard JM, Chervonsky AV. Intestinal fucose as a mediator of host-microbe symbiosis. J Immunol Baltim Md 1950. 2015;194:5588–93.

16. Zhang J, Sturla S, Lacroix C, Schwab C. Gut Microbial Glycerol Metabolism as an Endogenous Acrolein Source. mBio. 2018;9. doi:10.1128/mBio.01947-17.

17. Hughes ER, Winter MG, Duerkop BA, Spiga L, de Carvalho TF, Zhu W, et al. Microbial respiration and formate oxidation as metabolic signatures of inflammation-associated dysbiosis. Cell Host Microbe. 2017;21:208–19.

18. Pessione E. Lactic acid bacteria contribution to gut microbiota complexity: lights and shadows. Front Cell Infect Microbiol. 2012;2. doi:10.3389/fcimb.2012.00086.

19. Kröger A, Geisler V, Lemma E, Theis F, Lenger R. Bacterial fumarate respiration. Arch Microbiol. 1992;158:311–4.

20. El Aidy S, Derrien M, Merrifield CA, Levenez F, Doré J, Boekschoten MV, et al. Gut bacteria–host metabolic interplay during conventionalisation of the mouse germfree colon. ISME J. 2013;7:743–55.

21. Reese AT, Cho EH, Klitzman B, Nichols SP, Wisniewski NA, Villa MM, et al. Antibiotic-induced changes in the microbiota disrupt redox dynamics in the gut. eLife. 2018;7:e35987.

22. Garrote GL, Abraham AG, Rumbo M. Is lactate an undervalued functional component of fermented food products? Front Microbiol. 2015;6. doi:10.3389/fmicb.2015.00629.

23. Doo E-H, Chassard C, Schwab C, Lacroix C. Effect of dietary nucleosides and yeast extracts on composition and metabolic activity of infant gut microbiota in PolyFermS colonic fermentation models. FEMS Microbiol Ecol. 2017;93.

24. Waititu SM, Yin F, Patterson R, Yitbarek A, Rodriguez-Lecompte JC, Nyachoti CM. Dietary supplementation with a nucleotide-rich yeast extract modulates gut immune response and microflora in weaned pigs in response to a sanitary challenge. Anim Int J Anim Biosci. 2017;11:2156–64.

25. Sato N, Nakano T, Kawakami H, Idota T. In vitro and in vivo effects of exogenous nucleotides on the proliferation and maturation of intestinal epithelial cells. J Nutr Sci Vitaminol (Tokyo). 1999;45:107–18.

26. LeBlanc JG, Milani C, de Giori GS, Sesma F, van Sinderen D, Ventura M. Bacteria as vitamin suppliers to their host: a gut microbiota perspective. Curr Opin Biotechnol. 2013;24:160–8.

27. Raman M, Ahmed I, Gillevet PM, Probert CS, Ratcliffe NM, Smith S, et al. Fecal Microbiome and Volatile Organic Compound Metabolome in Obese Humans With Nonalcoholic Fatty Liver Disease. Clin Gastroenterol Hepatol. 2013;11:868-875.e3.

28. Kanehisa M. Toward understanding the origin and evolution of cellular organisms. Protein Sci Publ Protein Soc. 2019;28:1947–51.
